# Selective Mono- and Diamination of 2,6-Dibromopyridine for the Synthesis of Diaminated Proligands

**DOI:** 10.1021/acsomega.5c04396

**Published:** 2025-08-11

**Authors:** Alexander S. Underwood, Mark A. Botrous, Nate T. Lobb, Emmett M. Neal, Charlotte A. Richter, Mathieu A. Sleiman, Adrian B. Frye, Matthew Roberts, Clifford W. Padgett, Gary L. Guillet

**Affiliations:** † Department of Chemistry, 3628 Furman University, Greenville, South Carolina 29613, United States; ‡ Department of Biochemistry, Chemistry, and Physics, Georgia Southern University-Armstrong Campus, Savannah, Georgia 31419, United States

## Abstract

Selective mono- or diaminations of 2,6-dibromopyridine were performed using microwave irradiation with water as solvent in 2–2.5 h. The only significant difference between the syntheses was the inclusion of K_2_CO_3_ as base and CuI/DMPAO catalyst for the diaminations. The mono- and diaminations had approximately 7 and 2 g isolated yields, respectively. The monoaminated bromopyridines were attached to a TREN scaffolding molecule yielding novel ligands to support extended metal atom chain complexes.

## Graphical abstract

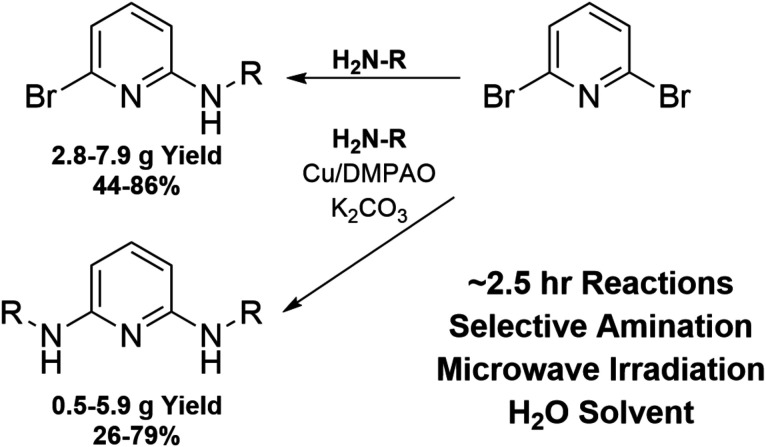

## Introduction

The continued interest in novel ligands to expand the library of extended metal atom chain complexes (EMAC) containing M–M bonds can be partly attributed to their potential in molecular electronics.^1–3^ EMACs, especially those with short M contacts, can have unique physical properties arising from direct magnetic exchange.^4,5^ Historically, 2,2′-dipyridylamine (dpa) (Figure 1a) has been the de facto ligand of choice to stabilize homo and heterotrimetallic EMACs. However, utilization of the same ligand resulted in the majority of these complexes taking on a consistent structural motif, namely a tetragonal ligand field around the trimetallic core along with two axial ligands (X) that are colinear with the M_3_ chain of the form M_3_(dpa)_4_X_2_. Our group has had success diverging from this pattern using 2,6-bis­(trimethylsilylamino)­pyridine (Figure 1b) to support a unique symmetric, axially vacant, trigonal triferrous EMAC of the form Fe_3_L_3_ with short Fe–Fe contacts (2.44 Å) for which there is no dpa congener.^6–8^ Following these prior results, a rational approach would be ligand modifications, leading to an expansion of known trigonal, triferrous EMACs. However, these efforts were hampered by limitations of the 2,6-silylaminopyridine platform. Hindrances include the limited commercially available alkyl/aryl chlorosilanes, the limited stability of the Fe_3_L_3_ complexes, which include high sensitivity to any protic or moderately donating solvent, air and moisture sensitivity, and low isolated crystalline yields (∼40%). Therefore, we endeavored to use the expansive library of primary amines to synthesize two classes of proligands to address the limitations experienced by the silylaminopyridine ligands.

**Figure 1:**
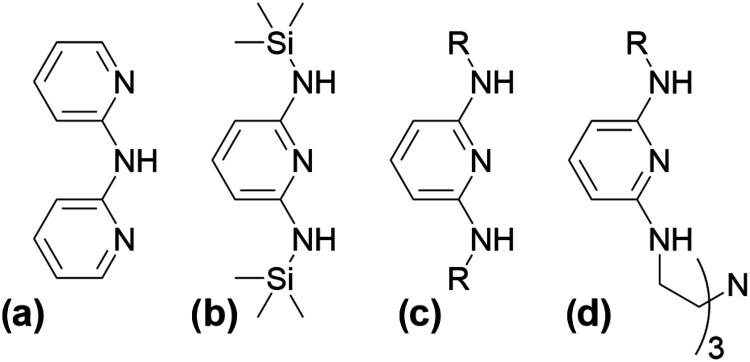
Structure of historically used EMAC supporting ligands: (a) 2,2′-dipyridylamine, (b) 2,6-bis­(trimethylsilylamino)­pyridine, and ligands developed herein, (c) 2,6-diaminopyridines and (d) scaffolded aminopyridines.

A potentially useful class of ligands is 2,6-diaminopyridines (Figure 1c). 2,6-Diaminopyridines have seen limited use as ligands with the only examples 2,6-bis­(methylamino)­pyridine supporting a tetranickel complex,^9^ and two homoleptic EMACs of the form (Bu_4_N)­[M_3_L_4_] where M is Cr­(II) or Ni­(II) and L is 2,6-di­(phenylamido)­pyridine.^10^ A related approach would be to incorporate a scaffolding molecule into the ligand, in effect combining all donor atoms into a single ligand (Figure 1d). This strategy could alleviate the entropic penalty in the formation of M_3_L_3_ type EMACs, effectively changing them to M_3_L. Such a strategy was successfully employed to stabilize homo and heterodimetallic complexes of Mn­(II), Fe­(II), and Co­(II) using tris­(2-aminoethyl)­amine (TREN) as the scaffold with trisamidopyridine or trisamidophosphine donor arms.^11,12^ There is also a report utilizing a scaffolded ligand that incorporates dpa based donor arms, albeit not resulting in an EMAC type structure.^13^

Key to successful synthesis of 2,6-diaminopyridines (henceforth **DAm**) and TREN scaffolded ligands (henceforth **TrAm**) is defining a protocol for the controlled amination of 2,6-dibromopyridine differentiating between asymmetric, monoamination leading to 2-bromo-6-aminopyridines (**Am**) and symmetric, diamination to form **DAm** derivatives. Scheme 1 describes the synthetic approach employed in this work. It does not rely upon Pd-catalyzed C–N bond formations^14,15^ taking advantage of the cost effectiveness and lessened immunotoxicity of Ullman type Cu catalysts. Cu has long been known to catalyze formation of these bonds^16^ but has often required harsh conditions limiting its application.^17^

**Scheme 1:**
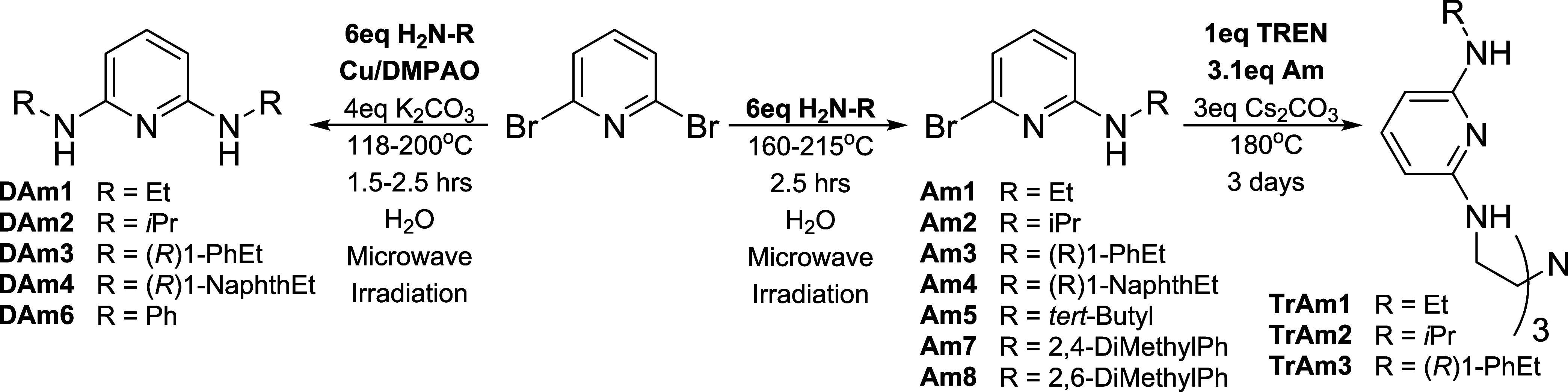
Summary of Synthetic Methods[Fn s1fn1]

a
**DAm6** synthesis did not include a Cu catalyst.

There has been a recent surge in novel Cu/L catalyst systems, with some capable of coupling arylhalides and secondary cyclic amines at room temperature, but these reactions require designer ligands, anhydrous conditions, strong bases, or long reaction times.^18,19^ There have also been studies investigating catalyzed asymmetric aminations of 2,6-dibromopyridine with primary and secondary amines, but these also rely on long reaction times (12–48 h) and conventional heating.^20,21^ The approach used herein to promote C–N bond formation was to use a commercially available Cu/L catalyst in combination with a microwave synthesizer under relatively mild conditions. There are examples of uncatalyzed^22^ and Cu catalyzed, microwave assisted C–C and C–heteroatom bond forming reactions^23,24^ as well as reactions that operate in milder conditions, for example with aqueous ammonia and Cu_2_O as a catalyst employing conventional heating^25,26^ or microwave irradiation,^27,28^ but these studies do not provide a generalized approach.

## Results and Discussion

Asymmetric, monoaminations were initially investigated with a range of primary alkylamines containing primary, secondary, or tertiary α-carbons. The monoaminated products were synthesized with high selectivity over the diaminated products using 1 equiv of 2,6-dibromopyridine (DBP), 6 equiv of the selected amine (70% ethylamine in H_2_O for **Am1**, isopropylamine for **Am2**, (*R*)-1-phenylethylamine for **Am3**, (*R*)-1-naphthylethylamine for **Am4**, and *tert*-butylamine for **Am5** ^29^) deionized H_2_O as solvent, and microwave irradiation for 2.5 h at 150–205 °C (see Supporting Information for details). It should be noted that for **Am5** the reaction time was increased to 3.0 h and complete conversion of DBP was not achieved. Precedent for the synthesis of **Am1**–**3** (Scheme 1) exists in the literature including under microwave conditions,^30–33^ however, these previous works report low yields, small scales, limited characterization, or the need for a Pd catalyst. Isolated yields ranged from 4.9–7.9 g (65–86%) despite the limits presented by using a microwave synthesizer. Because these asymmetric pyridines would be used in the synthesis of scaffolded proligands, substantial scales were required, and the reaction conditions were optimized for this purpose. **Am1**–**5** were purified by bulb-to-bulb distillation. Each compound was characterized by ^1^H and ^13^C NMR spectroscopies and HRMS (Figure 2). The major byproduct, if any, in these reactions was the diaminated product, which could be limited to ≤3.5% of the isolated product. Due to these compounds’ modest air sensitivity, they were stored in an inert atmosphere glovebox or a vacuum desiccator. Monoaminations were attempted with 2,2,2-trifluoroethylamine using the standard reaction conditions, including attempts with Cu/DMPAO catalyst, but all resulted in almost no conversion of DBP to products (Figure S31).

**Figure 2:**
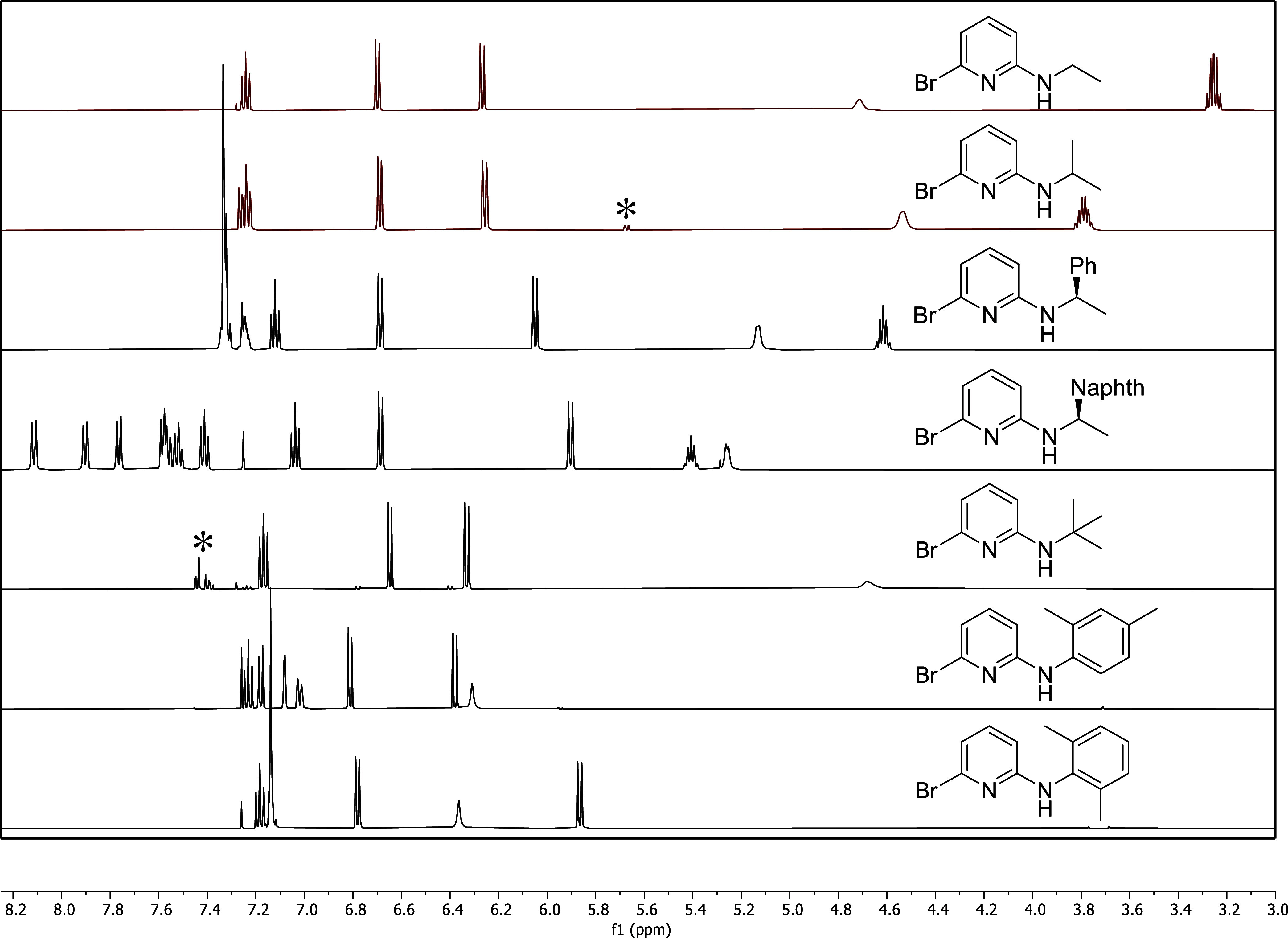
Partial ^1^H NMR spectra of **Am1**–**Am5** and **Am7**–**Am8** in chloroform-*d*. The asterisk denotes a signal from the diaminated byproduct for **Am2** and residual DBP for **Am5**.

To expand the substrate scope to arylamines, reactions involving aniline, 2,4-dimethylaniline, and 2,6-dimethylaniline were investigated under similar conditions to those used for alkylamines. For aniline, the asymmetric, monoaminated product could not be made to predominate under multiple temperature and time combinations because of facile formation of the symmetric, *diaminated* **DAm6**, along with the presence of residual DBP. When the reaction was performed at 200 °C for 2.5 h, **DAm6** was formed in 79% yield and approximately 5.5 g scale without the need for an exogenous base or catalyst. These results compare favorably against a conventionally heated, 4 h, 200 °C aniline/HCl melt^10^ and a 6 h, 225 °C microwave irradiated, CuI catalyzed reaction^32^ reporting yields of 65 and 43%, respectively. The diminished nucleophilicity of the bulkier anilines allowed for increased conversion of the monoaminated products. When either 2,4-dimethylaniline or 2,6-dimethylaniline were combined with DBP at high temperatures (190–215 °C) for 2.5 h the products **Am7** and **Am8** could be isolated in 55% and 43% yield respectively in 2–3 g scales (see Supporting Information for details).

The second amination of the pyridine ring generally proved to have a larger barrier for nucleophilic aromatic substitution prompting the need for a Cu/L catalyst.^34–36^ Considering this, we sought to identify a Cu/L catalyst to promote the synthesis of symmetric, diaminated **DAm** derivatives. A prior report suggested CuI alone could catalyze a range of diaminations of 2,6-dichloropyridine or 2,6-dibromopyridine using microwave irradiation, but required long microwave reaction times of up to 6 h.^32^ In our hands, this catalyst system did not attain the same efficacy. This is likely due to a difference microwave synthesizer model or slightly modified reaction conditions. Commercially available 2-(2,6-dimethylphenylamino)-2-oxoacetic acid (DMPAO), which was pioneered by Ma in 2012,^37^ efficiently catalyzes the N-arylation of acyclic secondary amines when combined with CuI. It was hypothesized that CuI/DMPAO, coupled with microwave irradiation, would promote symmetric, diaminations under similar conditions to those used for monoaminations. It should be noted, the synthetic targets in this work are also ligands and may coordinate to Cu, but their efficacy as Cu/L catalyst systems was not explicitly studied.^20^

In an effort to determine functional conditions for diaminations under microwave irradiation, the synthesis of **DAm3** was used as a test case. Standard conditions for comparison were 0.3 g scale of DBP, 140 °C, 45 min reaction time, and 3 mL of deionized H_2_O. Conversion was defined as the relative ratio of the **DAm3** to **Am3** derivatives, as measured by the peak area for the two meta-pyridyl peaks in the ^1^H NMR spectrum as well as the disappearance of peaks arising from DBP (7.38–7.49 ppm in chloroform-*d*). Initially, the necessity of the Cu/DMPAO catalyst system was investigated, and there was limited conversion if DMPAO was omitted (**DAm**/**Am** = 7/93). If the CuI or Cu/DMPAO was omitted, there was essentially exclusive conversion of **Am3** (Figure S32) with unreacted DBP observed in the spectrum.

The necessity of base was investigated using the standard conditions, and when using 4 equiv of K_2_CO_3_ the conversion was **DAm**/**Am** = 86/14, while omitting the base caused the conversion to shift to **DAm**/**Am** = 7/93 (Figure S33). The reaction conditions were then optimized for each **DAm** derivative (see Supporting Information). Each derivative could be synthesized by this catalyzed route in high purity and low to modest yield (25.6–46.0%), albeit on a smaller scale of 1.5 g of DBP compared to the monoaminated derivatives. **DAm1**–**2** could be purified by bulb-to-bulb distillation, whereas the others required column chromatography. Purity was confirmed by ^1^H and ^13^C NMR spectroscopies (Figure 3), and HRMS. Mass spectral analysis of a reaction residue for **DAm3** showed, in addition to the dominant signal with *m*/*z* = 318.17 assigned as [M + H], a peak of significant intensity at *m*/*z* = 411.17. This signal was tentatively assigned as a biarylether byproduct, presumably formed from the reaction of water and two equivalents of **Am3** (Figure S52). Crystals of **DAm3** were of sufficient quality for single crystal X-ray diffraction experiments. **DAm3** crystallized in the chiral (Sohncke) *P*2_1_2_1_2_1_ space group and data were collected with the Friedel pairs inequivalent. The Flack parameter = −0.1(2) indicates the absolute structure is properly described as *R*,*R*-2,6-bis­(1-phenylethylamino)­pyridine (Figure S53).

**Figure 3:**
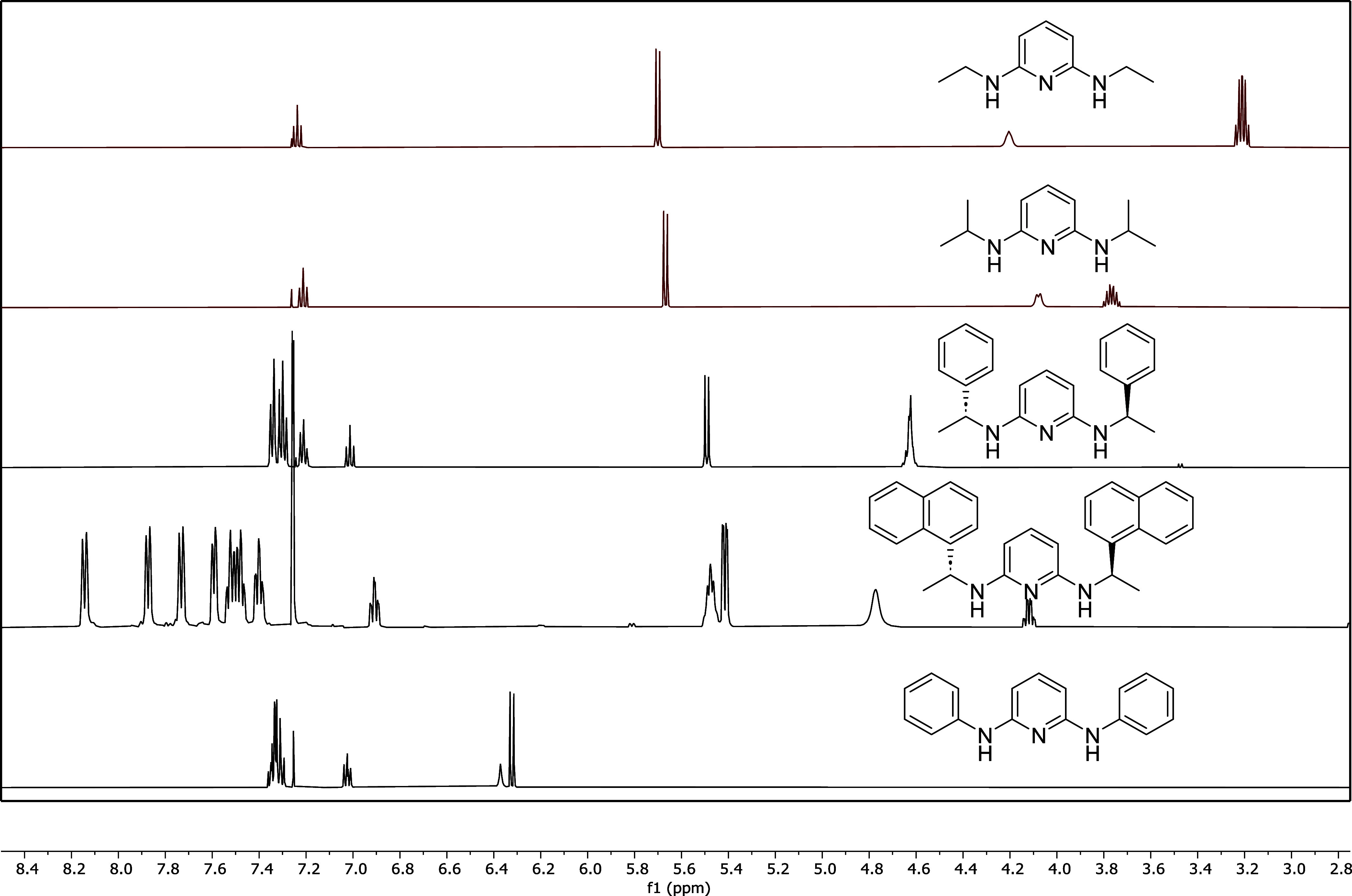
Partial ^1^H NMR spectra of **DAm1**–**4** and **DAm6** in chloroform-*d*.

For the arylamines 2,4-dimethylaniline and 2,6-dimethylaniline the ability of Cu/DMPAO to catalyze diaminations of DBP was investigated by running microwave irradiated reactions under identical conditions (190 °C for 2.5 h) with and without the Cu/DMPAO catalyst. ^1^H NMR spectra of the reaction residues were compared after a standard workup based on the peak integrations between DBP, the monoaminated, and diaminated compounds. For 2,4-dimethylaniline and 2,6-dimethylaniline the catalyst had a negligible impact on the extent of diamination, with almost identical degrees of the aminated produced from either set of conditions (Figures S34 and S35). Other studies investigating Cu catalyzed C–N bond forming reactions with DBP have observed challenges with arylamines compared to primary or secondary alkylamines. For example a study using Cu­(I)/TMEDA or Cu­(I)/DMEDA catalysts (TMEDA = *N*,*N*,*N*,*N*-tetramethylenediamine and DMEDA = *N,N*-dimethylethylenediamine) showed essentially no reactivity with aniline for mono- or diaminations and another study using either CuI or Pd­(PPh_3_)_4_ and microwave irradiation produced decreased yields with aniline and 2,6-dimethylaniline of 43 and 19%, relative to alkylamines in that report, after long reaction times (6 h) and elevated temperatures (225 °C).^21,32^ These results suggest unique Ullman type catalysts will have to be designed to efficiently couple arylamines with DBP.^38^

The utility of the asymmetric **Am** derivatives was shown by their attachment to the TREN scaffold in the synthesis of **TrAm1**–**3**. The synthetic approach followed a recent report by Cornia and co-workers, with minor adjustments, which described the synthesis of a scaffolded dpa variant that was later used to support a pentairon complex.^13,39^ **TrAm1**–**3** were formed by combination of 1 equiv of TREN with 3.25 equiv of the corresponding **Am** derivative and 3 equiv of Cs_2_CO_3_ (Scheme 1). The solvent free reactions were heated for 3 days at 180 °C with stirring under an argon atmosphere, solidifying and turning brown as the reaction progressed. After a base extraction was performed, the residues were then purified by silica gel column chromatography (see Supporting Information). **TrAm1**–**3** were characterized by ^1^H and ^13^C NMR spectroscopies and HRMS. Each derivative could be isolated in high purity and yield (57–70%) at a modest 2–3 g scale. The ^1^H NMR spectra in Figure 4 exhibit two distinct, broadened peaks for the N–H moieties (labeled a and a′) with downfield peaks indicative of the expected pyridyl hydrogen symmetry (labeled b and c). Each spectrum also showed two peaks for the TREN scaffold (labeled d and e), which in the case of **TrAm3** are clearly diastereotopic indicating that the chirality was maintained throughout the coupling reaction. Peaks labeled R refer to the alkyl or aryl moieties.

**Figure 4:**
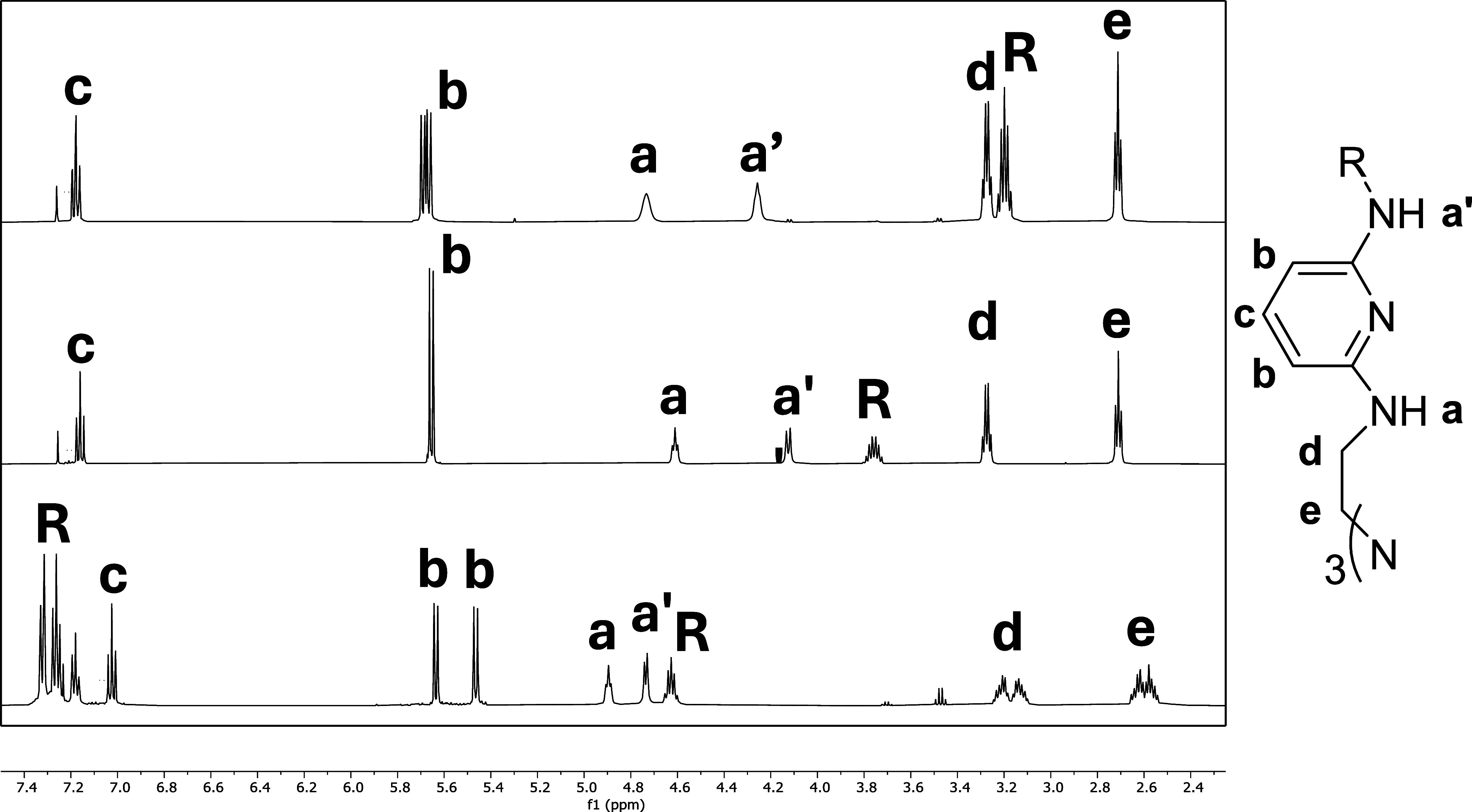
Comparison of partial ^1^H NMR spectra for **TrAm1** (*top*), **TrAm2** (*middle*), and **TrAm3** (*bottom*) in chloroform-*d*.

Reaction conditions and methods were determined to selectively mono- or diaminate 2,6-dibromopyridine with a set of primary alkylamines under microwave irradiation, with optimal yields requiring only small variations in the reaction conditions. All reactions were performed in water at 118–200 °C for approximately 2.5 h, and the diaminations generally only required addition of K_2_CO_3_ and a commercially available Cu/DMPAO catalyst system. The arylamines 2,4-dimethylaniline and 2,6-dimethylaniline could be monoaminted under the similar conditions as alkylamines. However, inclusion of the Cu/DMPAO catalyst was not effective to promote diamination. For aniline only the diaminated product could be isolated, without the need of base or catalyst. The asymmetric, monoaminated 2-bromo-6-aminopyridines could then be coupled to a TREN scaffold forming a useful set of proligands for applications in the synthesis of extended metal atom chain complexes. Notably, **TrAm3** is a chiral, multidentate ligand, opening avenues to investigate the impact of a chiral environment on M–M bonded EMACs. Between the diaminated and scaffolded proligands with similar substitution, the impact of a scaffolding molecule on EMAC formation, structure, and stability can be investigated.

## Supplementary Material



## Notes

Experimental procedures, characterization, spectra, and X-ray diffraction data (PDF)
