# Supplementary material for: Selective Mono- and Diamination of 2,6-Dibromopyridine for the Synthesis of Diaminated Proligands
Source: ACS Omega. 2025 Aug 11;10(32):36321–7. doi: 10.1021/acsomega.5c04396 (PMC12368628; doi:10.1021/acsomega.5c04396)
Supplement: Supplementary file 1 [file ao5c04396_si_001.pdf]

Supporting Information for:

Selective mono and diamination of 2,6-dibromopyridine for the synthesis of diaminated pro-ligands

Alexander S. Underwood,† Mark A. Botrous,† Nate T. Lobb,† Emmett M. Neal, † Charlotte A. Richter, † Mathieu A. Sleiman, † Adrian B. Frye,† Matthew Roberts,† Clifford W. Padgett,‡ Gary L. Guillet†,*

† Department of Chemistry, Furman University, Furman University, Greenville, SC 29613, USA

‡ Department of Biochemistry, Chemistry, and Physics, Georgia Southern University-Armstrong Campus, Savannah, GA 31419, USA

* Author to whom correspondence should be addressed.

Table of Contents

I.	General Information and Methods	Pg2
II.	Synthetic Procedures	Pg2
III.	^{1}H & ^{13}C NMR spectra	Pg10
IV.	IR Spectra of TrAm1-3	Pg27
V.	High-Res Mass Spectra	Pg28
VI.	X-Ray Crystallographic Data	Pg33
VII.	References	Pg35

I. General Information

Reagents were purchased by Fisher Chemical, VWR, or Sigma Aldrich and used without further purification, unless otherwise noted. CuI was stored in a vacuum desiccator when not in use. The deionized water was purified by a Purelab Chorus Water Purification System furnished by Elga LabWater. The microwave irradiated reactions were performed in a Discovery 2.0 Microwave Synthesizer made by CEM Corporation (Matthews, NC). Reactions were performed in 10, 35, or 100 mL glass reaction vessels with standard Teflon coated stir bars and caps. Deionized water was the solvent for all reactions. Specific rotation was measured on an Anton Parr (Ashland, VA) MCP 500 polarimeter using a sodium lamp (λ = 589 nm). Solutions were made in spectrograde THF and measured in a 1 decimeter polarimeter tube. NMR spectra were collected on a JEOL (Peabody, MA, USA) JNM-ECZR 500 MHz spectrometer. NMR solvents were purchased from Cambridge Isotope Labs and used as received. Spectra were referenced to the residual solvent peak of chloroform of 7.26 and 77.2 ppm in the ^{1}H and ^{13}C, respectively. If there was a conflict with this peak in the NMR spectrum, the TMS signal was used as a reference of 0.00 ppm. Infrared spectra were collected on a Perkin Elmer Spectrum 2 FTIR with ATR attachment (PerkinElmer, Shelton, MA, USA). High-resolution mass spectra were collected at the Mass Spectrometry Center at the University of South Carolina. All data was collected by direct injection using electrospray ionization in positive mode. Elemental analysis was performed by Midwest Microlabs (Indianapolis, IN, USA). All isolated compounds (**Am**, **DAm**, and **TrAm**) upon final purification, were stored in a Nex Gen inert atmosphere glovebox furnished by VAC Atmospheres Inc (Hawthorne, CA) using dinitrogen as the filler gas. The X-ray structure data were collected at 100 K on a Rigaku XtaLAB Synergy-i XRD diffractometer equipped with Cu-Ka radiation (1.54184 A) and processed using CrysAlis Pro (Rigaku,Tokyo, Japan) software. The structure was solved using direct methods and refined using full matrix least squares refinement using SHELXT and SHELXL in the Olex2 software package. Computer programs: *CrysAlis PRO*[1], *SHELXT2018/2*[2], *SHELXL2018/3*[3] and *OLEX2*.[4]

II. Synthetic Procedures

Synthesis of Am1 (2-bromo-6-ethylaminopyridine)

1 equivalent of dibromopyridine (8.2787 g, 34.947 mmol), 30 mL of water, and 6 equivalents of ethylamine in the form of a 70% v/v solution in water (16.56 mL) were charged into a 100 mL microwave vessel. The synthesis was run as a 2-stage method with stage one running at 145 °C for 45 minutes and stage two running at 160 °C for 1 hour and 45 minutes, for a total reaction time of 2.5 hours. The residue was extracted into 75 mL of DCM and washed with 75 mL of 0.1 M NaOH aqueous solution. The aqueous phase was extracted a second time with another 35 mL of DCM. The combined organic phase was dried with anhydrous magnesium sulfate. The organic phase was then filtered through a 2 cm plug of SiO_2. The solvents were removed from the filtrate on a rotary-evaporator. The product was then purified by Kugelrohr distillation at 115

°C and 40 mTorr. Am2 was isolated as a colorless, viscous liquid in 64.7% yield (4.547 g). The diaminated product, 2,6-diethylaminopyridine, was the only impurity at ~0.6% as analyzed by ^{1}H NMR. ^{1}H NMR (500 MHz, Chloroform-*d*) δ 7.22 (t, *J* = 7.9 Hz, 1H), 6.69 (d, *J* = 8.3 Hz, 1H), 6.26 (d, *J* = 8.3 Hz, 1H), 4.68 (br. s, 1H), 3.28 – 3.19 (m, 2H), 1.21 (t, *J* = 7.2 Hz, 3H). ^{13}C{^{1}H} NMR (126 MHz, Chloroform-*d*) δ 159.00, 140.34, 139.61, 115.60, 104.12, 37.03, 14.66. ESI-MS-TOF *m*/*z*: [M+H] calcd for $C_7H_{10}BrN_2$, 203.0001; found, 203.0000.

Synthesis of Am2 (2-bromo-6-isopropylaminopyridine)

1 equivalent of dibromopyridine (10.0118 g, 42.26 mmol), 20 mL of water, and 6 equivalents of isopropylamine (21.1 mL, 254 mmol) were charged into a 100 mL microwave vessel. The synthesis was run as a 2-stage method with stage one running at 150 °C for 45 minutes and stage two running at 165 °C for 1 hour and 45 minutes, for a total reaction time of 2.5 hours. The residue was extracted into 75 mL of DCM and washed with 75 mL of 0.1 M NaOH aqueous solution. The aqueous phase was extracted a second time with another 35 mL of DCM. The combined organic phase was dried with anhydrous magnesium sulfate. The solvents were removed at room temperature on a rotary-evaporator. The product was purified by Kugelrohr distillation at 115 °C and 40 mTorr. Am3 was isolated as a colorless, viscous liquid in 85.8% yield (7.7994 g). The diaminated product, 2,6-diisopropylaminopyridine, was the only impurity at 3.1% as analyzed by ^{1}H NMR. ^{1}H NMR (500 MHz, Chloroform-*d*) δ 7.24 (t, *J* = 8.0 Hz, 1H), 6.69 (d, *J* = 7.5 Hz, 1H), 6.25 (d, *J* = 8.2 Hz, 1H), 4.52 (br. s, 1H), 3.79p (m, 3.75-3.82, 1H), 1.22 (d, *J* = 6.4 Hz, 6H). ^{13}C NMR{^{1}H} (126 MHz, Chloroform-*d*) δ 158.34, 140.49, 139.63, 115.45, 104.44, 43.45, 22.87. ESI-MS-TOF *m*/*z*: [M+H] calcd for $C_8H_{12}BrN_2$, 217.0158; found, 217.0156. Anal. Calc (found) for $C_8H_{11}BrN_2$: H, 5.15 (5.11); C, 44.67 (44.60); N, 13.02 (13.18)%.

Synthesis of Am3 ((*R*)-2-bromo-6-(1-phenylethylamino)pyridine)

1 equivalent of dibromopyridine (8.9707 g, 37.88 mmol), 20 mL of water, and 6 equivalents of (*R*)-1-phenylethylamine (28.99 mL, 227.3 mmol) were charged into a 100 mL microwave vessel. The synthesis was run as a 2-stage method with stage one running at 150 °C for 30 minutes and stage two running at 175 °C for 2 hrs, for a total reaction time of 2.5 hours. The residue was extracted into 75 mL of DCM and washed with 75 mL of 0.1 M NaOH aqueous solution. The aqueous phase was extracted a second time with another 35 mL of DCM. The combined organic phase was dried with anhydrous magnesium sulfate. The solvents were removed at room temperature on a rotary-evaporator. Once the DCM was mostly removed, the temperature of the water bath was increased to 80 °C to remove the bulk of the excess amine. The remaining amine and a small amount of residual dibromopyridine were removed by a Kugelrohr distillation at 160 °C and 20 mTorr. The impurities were removed and the product could then be distilled on the same still at 180 °C and 20 mTorr. The product Am3 was isolated as a colorless, viscous liquid in 75.9% yield (7.973g). The diaminated product, 2,6-di((*R*)-1-phenylethylamino)pyridine, was not observed by ^{1}H NMR. $[\alpha]_{589}^{20}$ $+56.15°$ $([\boldsymbol{Am3}] = 0.0611\ g/mL, THF)$. ^{1}H NMR (500 MHz, Chloroform-*d*) δ 7.37 – 7.30 (m, 4H), 7.27 – 7.21 (m, 1H), 7.12 (t, *J* = 8.2 Hz, 1H), 6.69 (d, *J* = 7.7 Hz, 1H), 6.05 (d, *J* = 8.2 Hz, 1H), 5.13 (br. d, *J* = 6.1 Hz, 1H), 4.62 (p, *J* = 6.7 Hz,

1H), 1.53 (d, J = 6.8 Hz, 3H). $^{13}C\{^1H\}$ NMR (126 MHz, Chloroform-*d*) δ 158.18, 143.97, 140.21, 139.77, 128.91, 127.40, 125.91, 116.23, 104.62, 52.34, 24.47. ESI-MS-TOF *m*/*z*: [M+H] calcd for $C_{13}H_{14}BrN_2$, 277.0335; found, 277.0336. Anal. Calc (found) for $C_{13}H_{13}BrN_2$: H, 4.73 (4.76); C, 56.34 (56.43); N, 10.11 (11.03)%.

Synthesis of Am4 ((*R*)-2-bromo-6-(1-napthylethylamino)pyridine)

2,6-dibromopyridine (4.1478 g, 17.516 mmol), 30 mL of water, and 6 equivalents of (*R*)-1-napthylethylamine (16.8 mL, 105.096 mmol) were charged into a 100 mL microwave vessel. The synthesis was run as a 2-stage method with stage one running at 150 °C for 30 minutes and stage two running at 175 °C for 2 hours, for a total reaction time of 2.5 hours. The residue was extracted into 75 mL of DCM and washed with 75 mL of 0.1 M NaOH aqueous solution. The aqueous phase was extracted a twice more with 45 mL of DCM. The combined organic phase was dried with anhydrous magnesium sulfate. The solvents were removed at room temperature on a rotary-evaporator. The remaining amine and a small amount of residual dibromopyridine were removed by a Kugelrohr distillation at 175 °C and 80 mTorr. The product was then distilled using a bulb-to-bulb vacuum distillation employing two heating mantles at approximately 245 °C and 50 mTorr. The product was then isolated by running the residue through a silica gel plug with DCM as the eluant and removing the solvent on a rotary-evaporator. The product **Am4** was isolated as a light green, viscous liquid in 86.02% yield (4.9361)g. $[\alpha]_{589}^{22}$ $+57.0°$ $([\boldsymbol{Am4}] = 0.0539\ g/mL, THF)$. ^1H NMR (500 MHz, Chloroform-*d*) δ 8.12 (d, J = 8.4 Hz, 1H), 7.91 (d, J = 8.1 Hz, 1H), 7.77 (d, J = 8.2 Hz, 1H), 7.62 – 7.49 (m, 3H), 7.42 (t, J = 7.7 Hz, 1H), 7.05 (t, J = 7.9 Hz, 1H), 6.69 (d, J = 7.5 Hz, 1H), 5.91 (d, J = 8.2 Hz, 1H), 5.42 (app. p, J = 6.6 Hz, 1H), 5.27 (d, J = 5.9 Hz, 1H), 1.68 (d, J = 6.7 Hz, 3H). $^{13}C\{^1H\}$ NMR (126 MHz, Chloroform-*d*) δ 158.04, 140.25, 139.81, 138.91, 134.15, 130.68, 129.32, 128.00, 126.47, 125.90, 125.79, 122.53, 122.37, 116.29, 104.61, 48.50, 23.23. ESI-MS-TOF *m*/*z*: [M+H] calcd for $C_{17}H_{16}BrN_2$, 327.0491; found, 327.0493.

Synthesis of Am5 (2-bromo-6-(tert-butylamino)pyridine)

2,6-dibromopyridine (0.547 g, 2.31 mmol), 3.5 mL of water, and 6 equivalents of *tert*-butylamine (1.469 mL, 13.85 mmol) were charged into a 10 mL microwave vessel. The synthesis was run as a 3-stage method with stage one running at 160 °C for 15 minutes, stage at 195 °C for 30 minutes, and stage three running at 200 °C for 2.25 hours, for a total reaction time of 3 hours. The residue was extracted into 30 mL of DCM and washed with 30 mL of 0.1 M NaOH aqueous solution two times. The combines aqueous phase was then extracted with 30 mL of DCM. The combined organic phase was dried with anhydrous magnesium sulfate. The solvents were removed at room temperature on a rotary-evaporator. The residue was then redissolved in 30 mL of DCM and washed with 30 mL of 1% HCl two times. The combined aqueous phase was extracted with 30 mL of DCM and then the combined organic phase was washed with brine, dried with magnesium sulfate, and the solvent removed. The product was then separated by a Kugelrohr distillation at 90 °C and 40 mTorr for 45 minutes. The product **Am5** was isolated as a yellow liquid (0.287g) with modest air sensitivity and with a DBP impurity so a percent yield was not calculated. ^1H NMR (500 MHz, Chloroform-*d*) δ 7.18 (t, J = 7.7 Hz, 1H), 6.66 (d, J = 7.5 Hz, 1H), 6.33 (d, J = 8.3 Hz, 1H), 4.65 (s, 1H), 1.40 (s, 9H). $^{13}C\{^1H\}$ NMR (126 MHz,

Chloroform-*d*) δ 158.08, 139.88, 139.00, 115.01 106.49, 50.98, 29.20. ESI-MS-TOF *m*/*z*: [M+H] calcd for $C_9H_{14}BrN_2$, 229.0335; found, 229.0336.

Synthesis of Am7 (2-bromo-6-(2,4-dimethylphenylamino)pyridine)

1 equivalent of dibromopyridine (5.6902 g, 24.030 mmol), 30 mL of water, and 6 equivalents of 2,4-dimethylaniline (17.83 mL, 144.2 mmol) were charged into a 100 mL microwave vessel. The synthesis was run at 190 °C for 2.5 hours in a microwave synthesizer. The residue was extracted into 100 mL of DCM and washed with 100 mL of 1.5 M aqueous ammonia solution. The aqueous phase was extracted a second time with another 100 mL of DCM. The combined organic phase was dried with anhydrous magnesium sulfate and the solvents were removed at room temperature on a rotary-evaporator. The remaining 2,4-dimethylaniline and a small amount of residual dibromopyrinde were removed by a Kugelrohr distillation at 100 °C and 20 mTorr. The impurities were removed, and the residue was then distilled a second time at 150 °C and 20 mTorr. The product **Am7** was isolated as viscous yellow oil in 54.7% yield (3.6437 g) which crystallized upon standing. M.p. = 83.5-85.0°C. ^{1}H NMR (500 MHz, Chloroform-*d*) δ 7.28 – 7.14 (m, 2H), 7.08 (s, 1H), 7.02 (d, *J* = 8.1 Hz, 1H), 6.80 (d, *J* = 7.5 Hz, 1H), 6.40 – 6.31 (m, 2H), 2.32 (s, 3H), 2.21 (s, 3H). ^{13}C{^{1}H} NMR (126 MHz, Chloroform-*d*) δ 157.83, 140.33, 139.88, 135.68, 134.73, 133.21, 131.97, 127.64, 124.96, 117.20, 104.80, 21.02, 18.00. ESI-MS-TOF *m*/*z*: [M+H] calcd for $C_{13}H_{14}BrN_2$, 277.0335; found, 277.0336.

Synthesis of Am8 (2-bromo-6-(2,6-dimethylphenylamino)pyridine)

1 equivalent of dibromopyridine (5.5507g, 23.4405 mmol), 30 mL of water, and 6 equivalents of 2,6-dimethylaniline (17.16 mL, 140.6 mmol) were charged into a 100 mL microwave vessel. The synthesis was run as a triple stage method with stage one running at 190°C for 30 minutes, stage two running at 205°C for one hour, and stage three running at 215°C for one hour for a total of 2.5 hours. The residue was extracted into 75 mL of DCM and washed with 75 mL of a 1.5M aqueous solution of $NH_4^+OH^-$. The aqueous phase was extracted a second time with 50 mL of DCM. The combined organic phase was dried with anhydrous magnesium sulfate. The solvents were removed at room temperature with a rotary evaporator. Excess 2,6-dimethylaniline was removed using Kugelrohr distillation at 125°C and 60 mTorr for approximately 45 minutes. The residue was purified with a second Kugelrohr distillation at 185°C and 60 mTorr to distill the product as a viscous orange liquid in 43.9% yield (2.8129 g) which crystalized upon sitting. M.p. = 88.9-92.0 °C. ^{1}H NMR (500 MHz, Chloroform-*d*) δ 7.18 (t, *J* = 7.9 Hz, 1H), 7.14 (d, *J* = 1.0 Hz, 3H), 6.78 (d, *J* = 7.6 Hz, 1H), 6.36 (s, 1H), 5.87 (d, *J* = 8.2 Hz, 1H), 2.22 (s, 6H). ^{13}C{^{1}H} NMR (126 MHz, Chloroform-*d*) δ 158.08, 140.39, 140.09, 137.01, 135.57, 128.78, 127.44, 116.77, 103.73, 18.46. ESI-MS-TOF *m*/*z*: [M+H] calcd for $C_{13}H_{14}BrN_2$, 277.0335; found, 277.0336.

Synthesis of DAm1, 2,6-di(ethylamino)pyridine

To a 35 mL microwave vessel was charged 2,6-dibromopyridine (1.6392g, 6.943 mmol), K_2CO_3 (3.84g, 27.8 mmol), CuI (132 mg, 0.694 mmol), and DMPAO (268 mg, 1.39 mmol). 10 mL of water were added to the reaction and then 70% ethylamine in H_2O solution (3.29 mL, 41.7 mmol) was added via micropipettor. The reaction was then heated to 125 °C for 90 min in the microwave synthesizer. After cooling, the reaction residue was extracted into 50 mL of DCM and washed with 50 mL of 0.1 M NaOH. The aqueous phase was extracted with an additional 25 mL of DCM. The combined organics were then washed with 25 mL of 0.1 M NaOH. The organic phase was dried with $MgSO_4$, filtered, and the solvent removed on a rotary-evaporator. The dark brown residue was then distilled in a Kugelrohr still at 130 °C and 70 mTorr to yield a viscous yellow oil in 45.6% yield (0.5203g). ^{1}H NMR (500 MHz, Chloroform-*d*) δ 7.24 (t, J = 7.9 Hz, 1H), 5.70 (d, J = 7.9 Hz, 2H), 4.20 (br. t, J = 5.7 Hz, 2H), 3.21 (qd, J = 7.1, 5.5 Hz, 4H), 1.21 (t, J = 7.2 Hz, 6H). $^{13}C\{^1H\}$ NMR (126 MHz, Chloroform-*d*) δ 158.40, 139.14, 94.33, 37.00, 15.03. ESI-MS-TOF *m/z*: [M+H] calcd for $C_9H_{16}N_3$, 166.1339; found, 166.1339.

Synthesis of DAm2, 2,6-di(isoprppylamino)pyridine

To a 35 mL microwave vessel was charged 2,6-dibromopyridine (1.7101g, 7.243 mmol), K_2CO_3 (4.00 g, 28.9 mmol), CuI (138 mg, 0.724 mmol), and DMPAO (279 mg, 1.44 mmol). 15 mL of water were added to the reaction and then diisopropylamine (3.72 mL, 43.4 mmol) was added via micropipettor. The reaction was then heated to 118°C for 2.5 hr in the microwave synthesizer. After cooling, the reaction residue was extracted into 50 mL of DCM and washed with 50 mL of 0.1 M NaOH. The aqueous phase was extracted with an additional 25 mL of DCM. The combined organics were then washed with 25 mL of 0.1 M NaOH. The organic phase was dried with $MgSO_4$, filtered, and the solvent removed on a rotary-evaporator. The dark brown residue was then distilled in a Kugelrohr still at 150 °C and 80 mTorr to yield a viscous yellow oil in 46.0% yield (0.644 g). ^{1}H NMR (500 MHz, Chloroform-*d*) δ 7.21 (t, J = 7.9 Hz, 1H), 5.67 (d, J = 7.9 Hz, 2H), 4.08 (br. d, J = 8.0 Hz, 2H), 3.76 (dspt, J = 7.7, 1.3 Hz, 2H), 1.19 (d, J = 6.5 Hz, 12H). ^{13}C NMR (126 MHz, Chloroform-*d*) δ 157.77, 139.08, 94.48, 43.11, 23.25. ESI-MS-TOF *m/z*: [M+H] calcd for $C_{11}H_{20}N_3$, 194.1652; found, 194.1653.

Synthesis of DAm3, *R,R*-2,6-di(1-phenylethylamino)pyridine

2,6-Dibromopyridine (1.7292 g, 7.324 mmol), K_2CO_3 (4.04g, 29.3 mmol), CuI (139 mg, 0.732 mmol), DMPAO (283 mg, 1.47 mmol), 15 mL of water, and 6 equivalents of (*R*)-1-phenyltheyamine (5.61 mL, 43.9 mmol) were combined in a 35 mL microwave reaction vessel. The reaction was run at 130 °C for 90 min reaching a maximum pressure of ~50 psi within 3 minutes and decreasing for the remainder of the reaction. After the reaction cooled, it was extracted with 50 mL of DCM and 50 mL of 0.1 M NaOH. The aqueous phase was extracted two additional times with 15 mL of DCM. The combined organics were washed with 50 mL of 0.1 M NaOH. The organic phase was then dried with $MgSO_4$, filtered, and the DCM removed on a rotary evaporator. The residue had residual amine removed using a Kugelrohr still at 100°C

and 100 mTorr for ~1hr. The distillate was removed and the residue was then purified by column chromatography with silica gel. The column was loaded and the plug made with DCM. A volume of DCM was run through the column approximately equal to the height of the column. The eluent was then changed to 3/1 hexane/ethyl acetate to elute DAm3. The solvent was removed, and the resulting yellow oil was dried at 80 °C for 2 hours resulting in a 25.6% yield (0.5957 g). X-ray quality crystals were afforded by recrystallization from Et_2O at -30 °C. M.p. = 92.0 – 92.5 °C. ^{1}H NMR (500 MHz, Chloroform-*d*) δ 7.35 – 7.27 (m, 8H), 7.25 – 7.19 (m, 2H), 7.03 (t, *J* = 7.9 Hz, 1H), 5.50 (d, *J* = 8.0 Hz, 2H), 4.63 (m, 4H), 1.48 (d, *J* = 6.7 Hz, 6H). $^{13}C\{^1H\}$ NMR (126 MHz, Chloroform-*d*) δ 157.36, 145.36, 139.20, 128.65, 126.92, 125.98, 95.59, 24.58. ESI-MS-TOF *m/z*: [M+H] calcd for $C_{21}H_{24}N_3$, 318.1965; found, 318.1965.

Synthesis of DAm4, *R*-2,6-di(1-naphthylethylamino)pyridine

2,6-Dibromopyridine (1.5782 g, 6.6845 mmol), K_2CO_3 (3.6420g, 26.353 mmol), CuI (171 mg, 0.899 mmol), DMPAO (274 mg, 1.42 mmol), 18 mL of water, and 6 equivalents of (*R*)-1-naphtheylamine (6.42 mL, 40.11 mmol) were combined in a 35 mL microwave reaction vessel. The reaction was run at 125 °C for 2 hr. After the reaction cooled, it was extracted with 50 mL of DCM and 50 mL of 0.1 M NaOH. The aqueous phase was extracted with an additional two times with 15 mL of DCM. The combined organics were washed with 50 mL of brine. The organic phase was then dried with $MgSO_4$, filtered, and the DCM removed on a rotary evaporator. The residue had residual amine removed using a Kugelrohr still at 175°C and 90 mTorr for ~1hr. The distillate was removed, and the residue was then purified by column chromatography with silica gel. The column was loaded and the plug made with DCM. A volume of DCM was run through the column approximately equal to the height of the column. The eluent was then changed to 3/1 hexane/ethyl acetate to elute **DAm4**. The solvent was removed, and the resulting gray-green powder was dried at 80°C for 2 hours resulting in a 39.80% yield (1.1109g). M.p. = 88.0-90.0 °C. ^{1}H NMR (500 MHz, Chloroform-*d*) δ 8.15 (d, *J* = 8.4 Hz, 2H), 7.88 (d, *J* = 8.0 Hz, 2H), 7.74 (d, *J* = 8.1 Hz, 2H), 7.60 (d, *J* = 7.2 Hz, 2H), 7.56 – 7.45 (m, 5H), 7.40 (t, *J* = 7.7 Hz, 2H), 6.91 (t, *J* = 7.9 Hz, 1H), 5.48 (p, *J* = 6.6 Hz, 2H), 5.42 (d, *J* = 7.9 Hz, 2H), 4.79 (s, 2H), 1.58 (d, *J* = 6.7 Hz, 6H). $^{13}C\{^1H\}$ NMR (126 MHz, Chloroform-*d*) δ 157.20, 140.43, 139.25, 134.09, 130.93, 129.13, 127.54, 126.19, 125.89, 125.57, 122.95, 122.35, 95.67, 47.99, 23.13. ESI-MS-TOF *m/z*: [M+H] calcd for $C_{29}H_{28}N_3$, 418.2278; found, 418.2278.

Synthesis of DAm6, 2,6-di(phenylamino)pyridine

To a 100 mL microwave vessel was charged 2,6-dibromopyridine (6.3669g, 26.89 mmol), 30 mL of water, and then 6 equivalents of aniline (14.729 mL, 161.32 mmol) was added via micropipettor. The reaction was then heated to 200 °C for 150 min in the microwave synthesizer. After cooling, the reaction residue was extracted into 75 mL of DCM and washed with 75 mL of 1% HCl three times, then washed with 75 ml 1.5 M ammonia solution. The ammonia aqueous phase was extracted with an additional 25 mL of DCM. The combined organic phases were dried with $MgSO_4$, filtered, and the solvent removed on a rotary-evaporator. The dark residue

was then run through a silica gel plug using DCM as eluent to remove unwanted byproducts. The solvent was removed from the resulting yellow solution using the rotary-evaporator. The residue was then distilled in a Kugelrohr still at 120 °C for 45 mins at 70 mTorr to yield a viscous yellow oil. The oil crystallized upon sitting and was dried on the Schlenk line at 70 °C for 2.5 hours resulting in a yellow powder in 79.43% yield (5.5813g). M.p. = 98.0 – 100.0 °C. ^{1}H NMR (500 MHz, Chloroform-*d*) δ 7.39 – 7.28 (m, 9H), 7.03 (tt, *J* = 6.9, 1.5 Hz, 2H), 6.38 (s, 2H), 6.33 (d, *J* = 8.0 Hz, 2H). ^{13}C NMR{^{1}H} (126 MHz, Chloroform-*d*) δ 154.98, 140.64, 139.34, 129.14, 122.41, 120.26, 98.94. ESI-MS-TOF *m/z*: [M+H] calcd for $C_{17}H_{16}N_3$, 262.1339; found, 262.1339.

Synthesis of TrAm1

To a 100 mL Schlenk flask was added **Am1** (3.00 g, 14.9 mmol) along with cesium carbonate (4.86 g, 14.9 mmol). The atmosphere was removed from the flask with vacuum and replaced with argon. TREN (685 µL, 4.52 mmol) was added to the flask with a counter flow of Ar. The reaction was stirred at 180 °C for 3 days and during the reaction it turned into a mostly solid brown mass. After cooling, the residue was extracted into 80 mL of DCM and washed with 70 mL of 0.1 M NaOH solution. The aqueous phase was then extracted with an additional 15 mL of DCM. The combined organic phase was dried with magnesium sulfate, filtered, and the solvent removed under reduced pressure. The residue was then purified by silica gel chromatography. The column was initially loaded and un with DCM until the residual Am1 was eluted. The eluent was then changed to ethyl acetate/ethanol (20/1 v:v) with 3% triethylamine to elute the product **TrAm1** off the column. The combined fractions had their solvent removed under reduced pressure and the **TrAm1** was then dried at 125 °C under vacuum for 2 hours to remove residual solvent. The compound was isolated as a tacky brown solid in 57.6% yield (1.32 g). ^{1}H NMR (500 MHz, Chloroform-*d*) δ 7.18 (t, *J* = 7.9 Hz, 3H), 5.69 (d, *J* = 7.6 Hz, 3H), 5.66 (d, *J* = 7.8 Hz, 3H), 4.73 (br. s, 3H), 4.25 (br. s, 3H), 3.27 (q, *J* = 6.5 Hz, 6H), 3.19 (spt, *J* = 7.1 Hz, 6H), 2.71 (t, *J* = 5.9 Hz, 6H), 1.19 (t, *J* = 7.2 Hz, 9H). ^{13}C{^{1}H} NMR (126 MHz, Chloroform-*d*) δ 158.38, 158.33, 139.06, 95.83, 93.96, 53.66, 40.15, 37.13, 15.08. ESI-MS-TOF *m/z*: [M+H] calcd for $C_{27}H_{43}N_{10}$, 507.3667; found, 507.3667.

Synthesis of TrAm2

To a 100 mL Schlenk flask inside an inert atmosphere glovebox was added **Am2** (4.745 g, 22.06 mmol). The flask was moved to a Schlenk line and cesium carbonate (6.53 g, 20.1 mmol) and TREN (1.000 mL, 6.685 mmol) were added under counter Ar flow. The reaction was stirred at 180 °C for 3 days and during the reaction it turned into a mostly solid brown mass. After cooling, the residue was extracted into 75 mL of DCM and washed with 75 mL of 0.1 M NaOH solution. The aqueous phase was then extracted with an additional 25 mL of DCM. The combined organic phase was dried with magnesium sulfate, filtered, and the solvent removed under reduced pressure. The residue was then purified by silica gel chromatography. The column was initially loaded with DCM until the residual Am2 was eluted. The eluent was then changed to ethyl acetate/ethanol/10% aq. NH_4OH (60/1/1 v:v) to move the product **TrAm2** off

the column. The combined fractions had their solvent removed under reduced pressure and the **TrAm2** was then dried at 100 °C under vacuum for 1hr to remove residual solvent. The compound was isolated as a brown, tacky solid in 57.8% yield (2.1718 g). ^{1}H NMR (500 MHz, Chloroform-*d*) δ 7.16 (t, *J* = 7.9 Hz, 3H), 5.66 (app. d, *J* = 7.9 Hz, 6H), 4.61 (t, *J* = 5.7 Hz, 3H), 4.13 (d, *J* = 8.0 Hz, 3H), 3.81 – 3.71 (m, 3H), 3.28 (q, *J* = 6.0 Hz, 6H), 2.70 (t, *J* = 6.3 Hz, 6H), 1.18 (d, *J* = 6.4 Hz, 19H). ^{13}C{^{1}H} NMR (126 MHz, Chloroform-*d*) δ 158.48, 157.73, 138.96, 95.46, 94.54, 53.69, 43.17, 40.15, 23.26. ESI-MS-TOF *m*/*z*: [M+H] calcd for $C_{30}H_{49}N_{10}$, 549.4136; found, 549.4136.

Synthesis of TrAm3

To a 100 mL Schlenk flask was added Am3 (5.4020 g, 19.48 mmol) along with cesium carbonate (5.86 g, 17.9 mmol). The atmosphere was removed from the flask with vacuum and replaced with argon. TREN (969 µL, 6.00 mmol) was added to the flask with a counter flow of Ar. The reaction was stirred at 185 °C for 3.5 days and during the reaction it turned into a mostly solid brown mass. After cooling, the residue was extracted into 30 mL of DCM and washed with 15 mL of 0.1 M NaOH solution. The aqueous phase was then extracted with an additional 10 mL of DCM. The combined organic phase was dried with magnesium sulfate and the solvent removed under reduced pressure. The residue was then purified by silica gel chromatography. The column was initially loaded with DCM and the residual Am3 was eluted. The eluent was changed to ethyl acetate, ethanol, triethylamine (100/5/1 v:v) to move the product **TrAm3** off the column. The combined fractions had their solvent removed under reduced pressure. The **TrAm3** was then dried at 125 °C and 20 mTorr for 2 hours to remove residual solvent. The compound was isolated as a brown solid in 69.8% yield (3.0774 g). ^{1}H NMR (500 MHz, Chloroform-*d*) δ 7.40 – 7.24 (m, 12H), 7.24 – 7.14 (m, 3H), 7.03 (t, *J* = 7.9 Hz, 3H), 5.64 (d, *J* = 7.9 Hz, 3H), 5.48 (d, *J* = 7.9 Hz, 3H), 4.93 (br. t, *J* = 5.7 Hz, 3H), 4.73 (br. d, *J* = 6.2 Hz, 3H), 4.63 (p, 3H), 3.18 (m, 6H), 2.68 – 2.54 (m, 6H), 1.47 (d, 9H). ^{13}C{^{1}H} NMR (126 MHz, Chloroform-*d*) δ 158.23, 157.32, 145.54, 139.06, 128.61, 126.83, 125.97, 96.05, 94.91, 53.40, 52.08, 40.00, 24.67. ESI-MS-TOF *m*/*z*: [M+H] calcd for $C_{45}H_{55}N_{10}$, 735.4606; found, 735.4614.

III. NMR Spectra

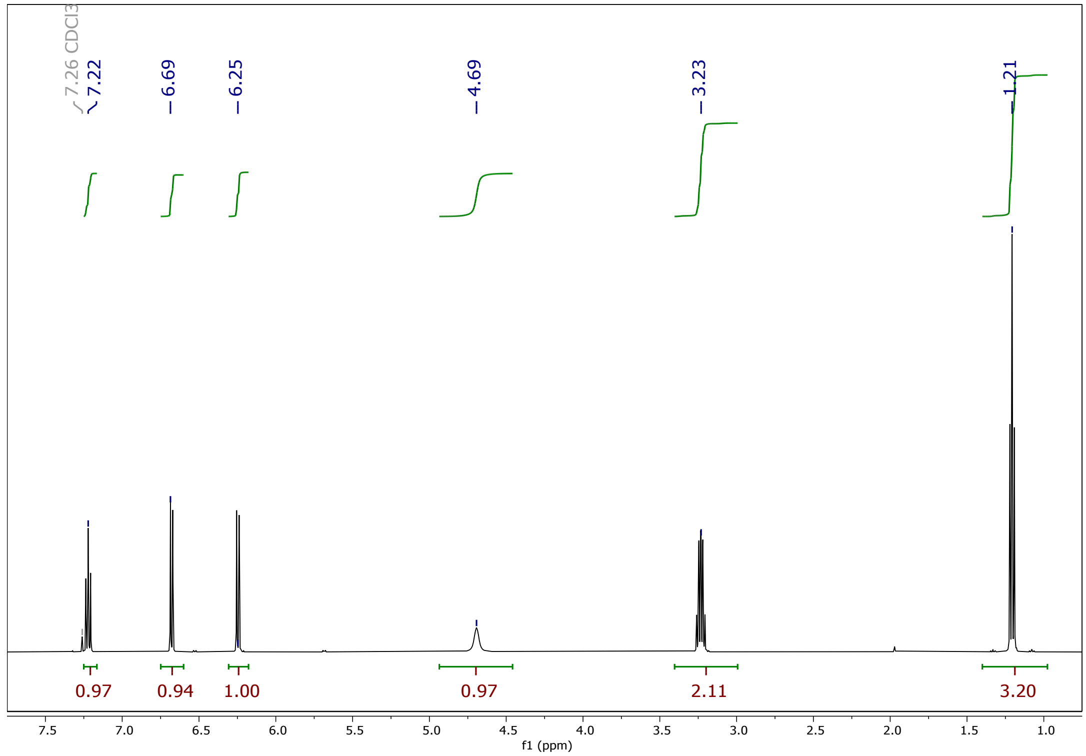

Figure S1. ^{1}H NMR spectrum of **Am1** in $CDCl_3$.

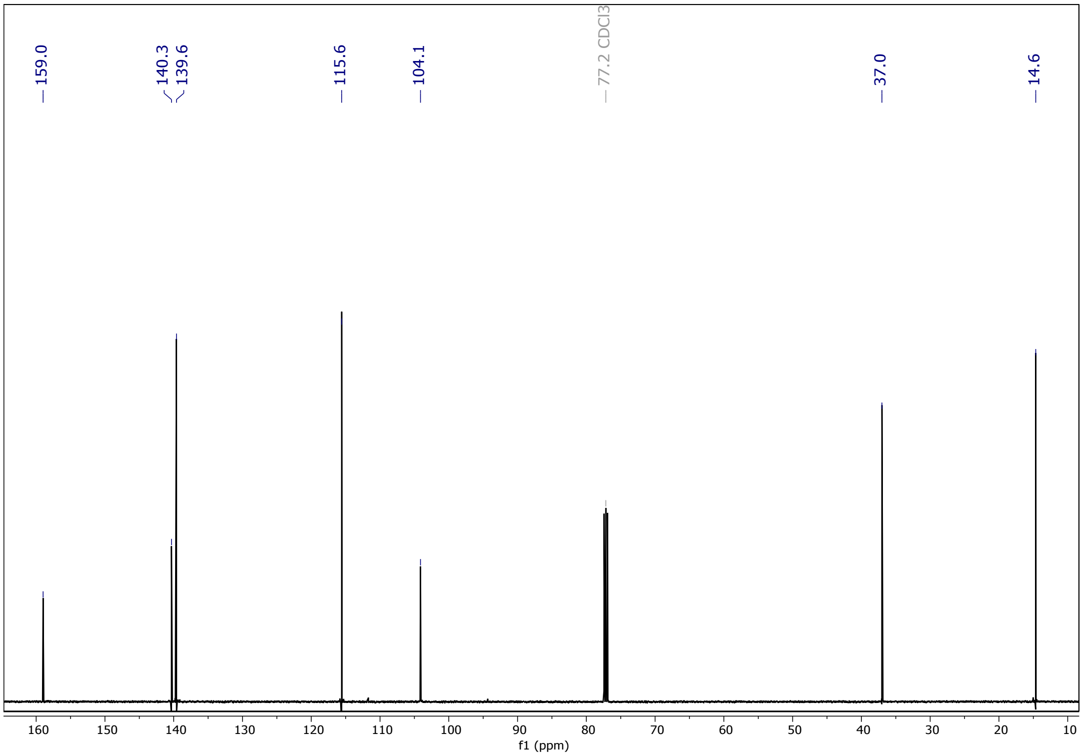

Figure S2. ^{13}C NMR spectrum of **Am1** in $CDCl_3$.

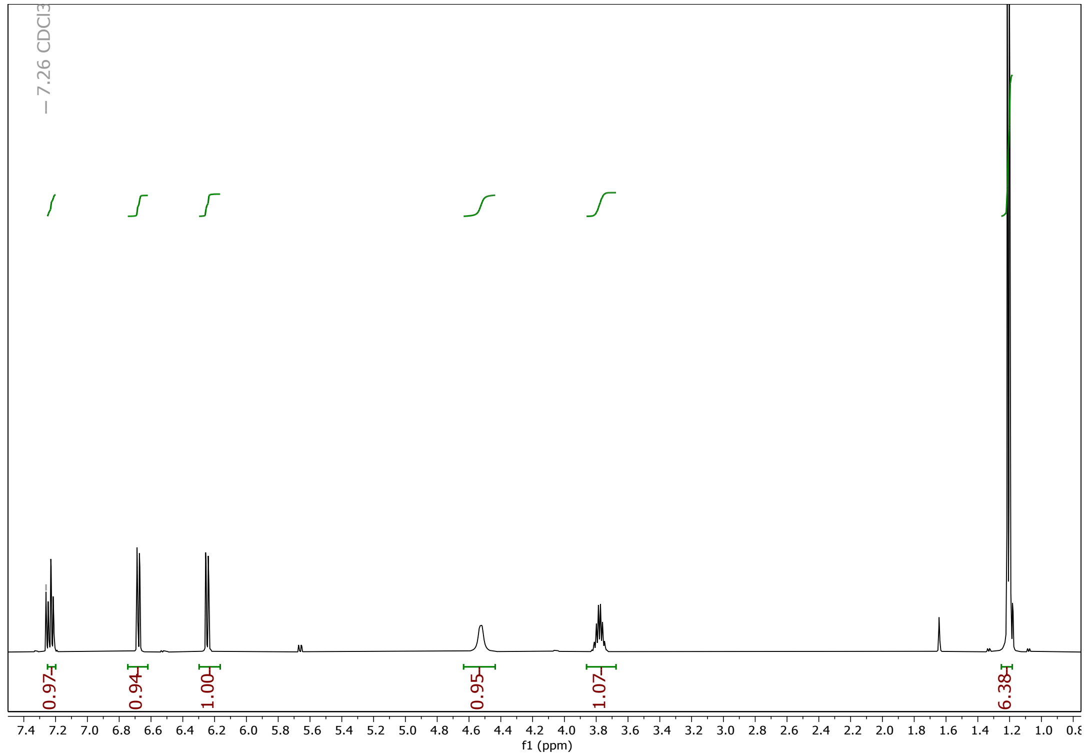

Figure S3. ^{1}H NMR spectrum of **Am2** in $CDCl_3$.

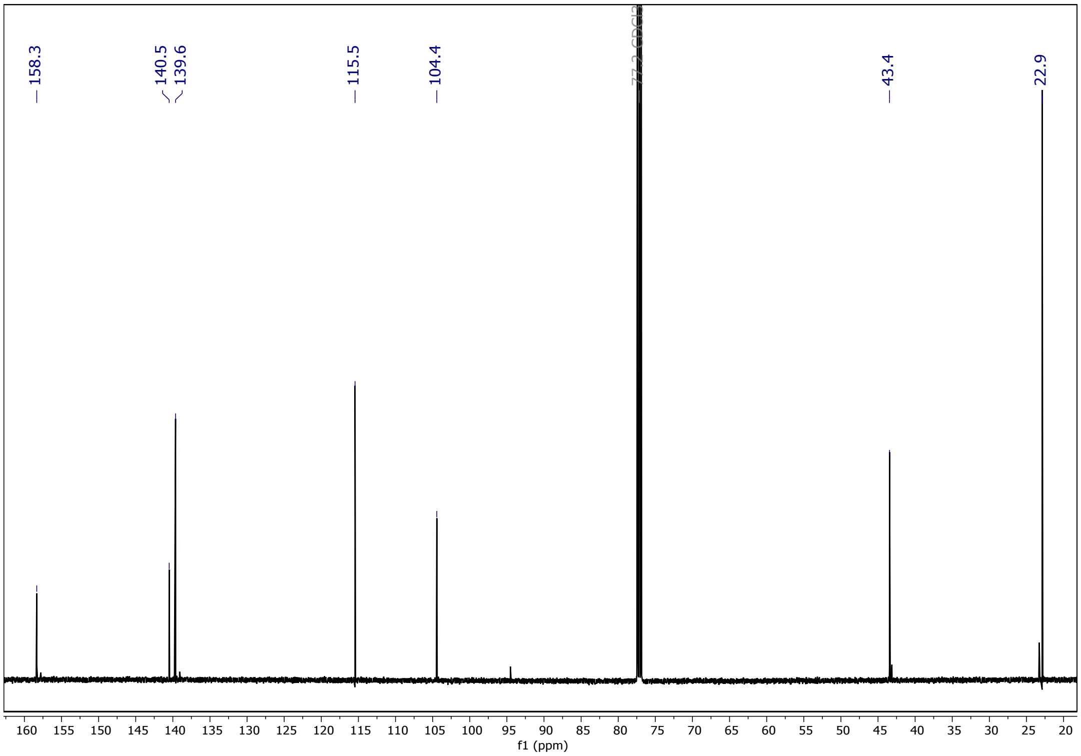

Figure S4. ^{13}C NMR spectrum of **Am2** in $CDCl_3$.

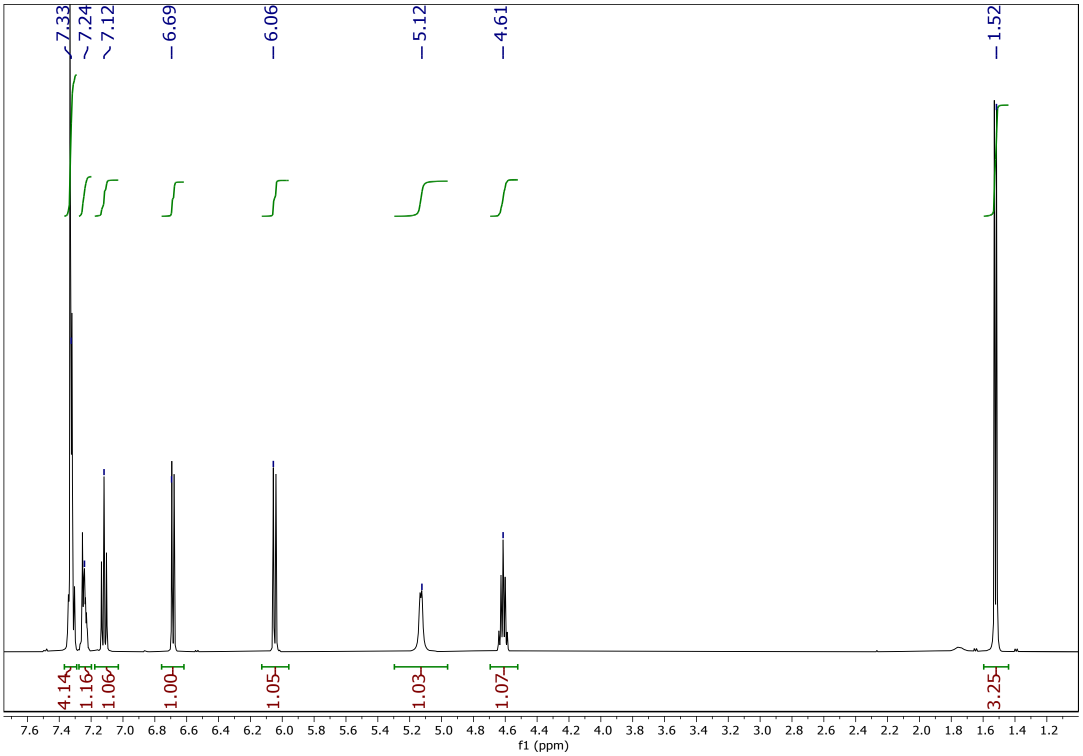

Figure S5. ^{1}H NMR spectrum of **Am3** in $CDCl_3$.

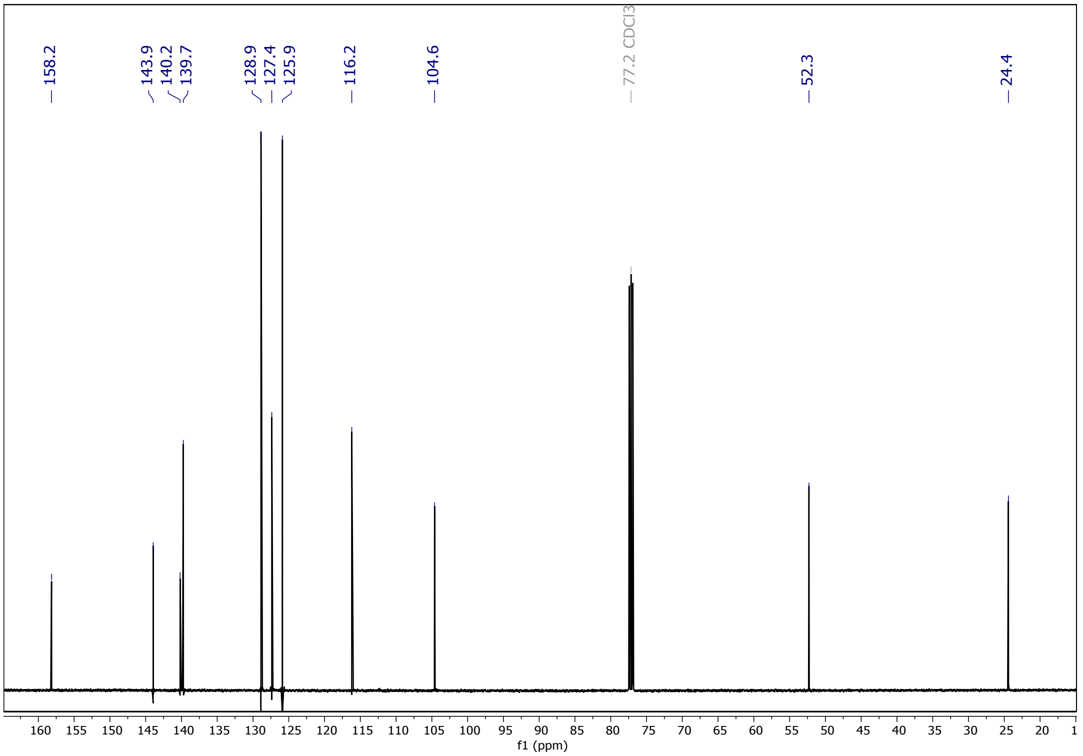

Figure S6. ^{13}C NMR spectrum of **Am3** in $CDCl_3$.

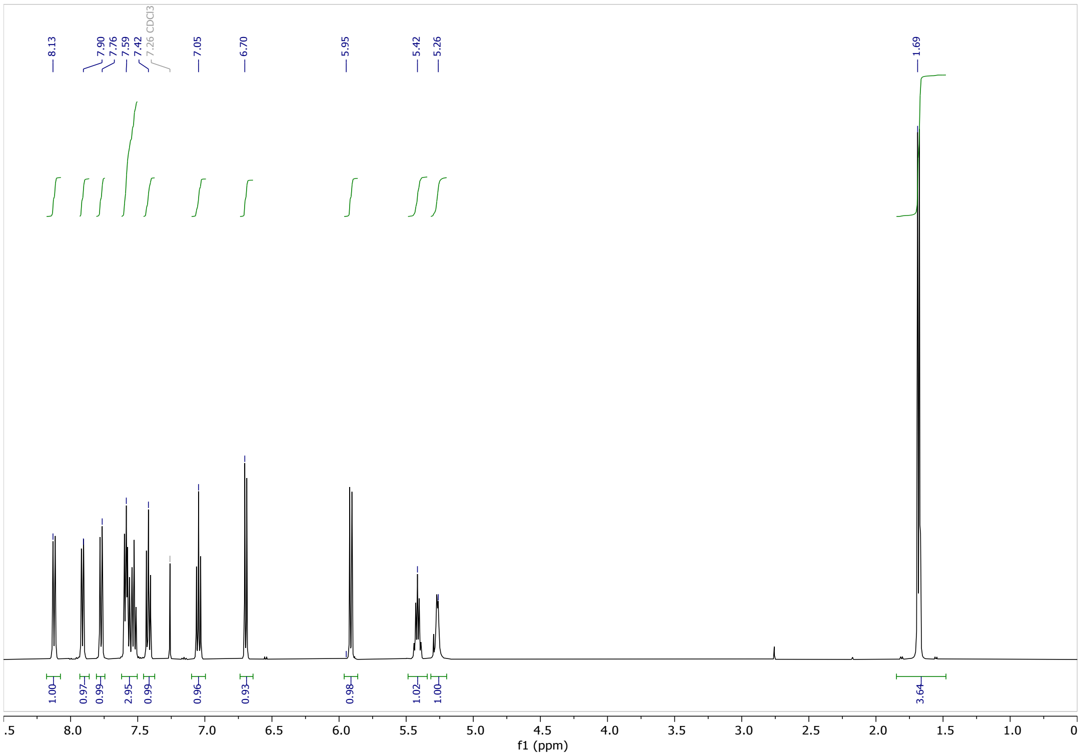

Figure S7. ^{1}H NMR spectrum of **Am4** in $CDCl_3$.

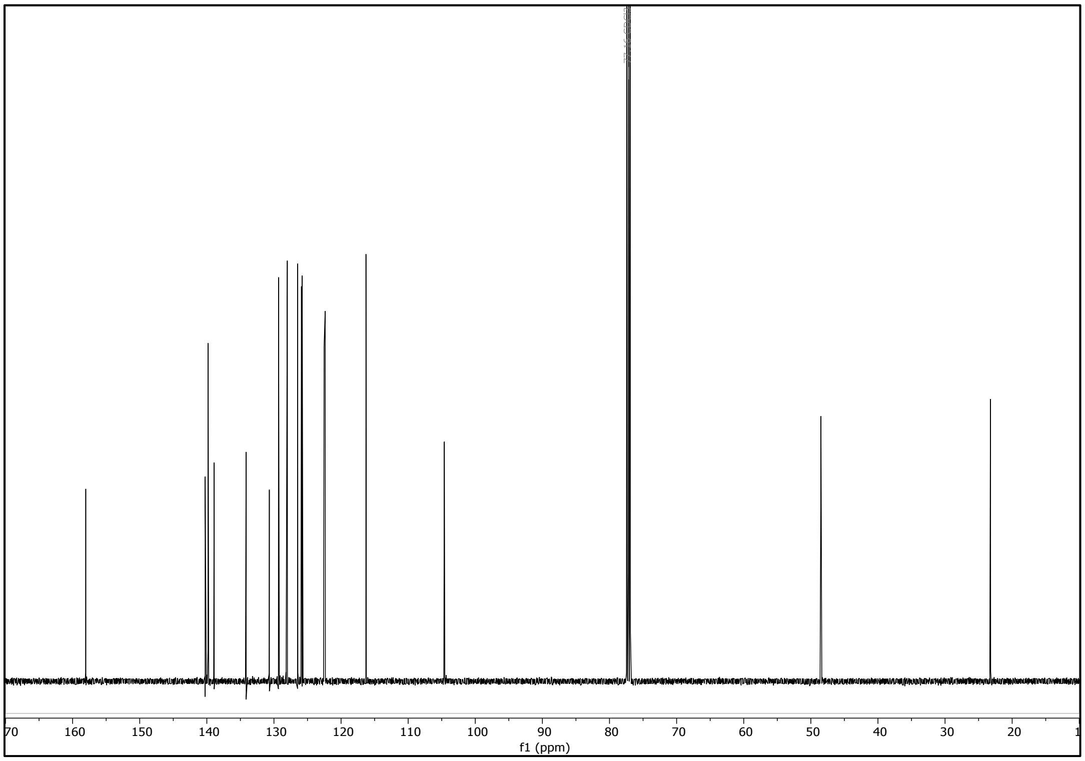

Figure S8. ^{13}C NMR spectrum of **Am4** in $CDCl_3$.

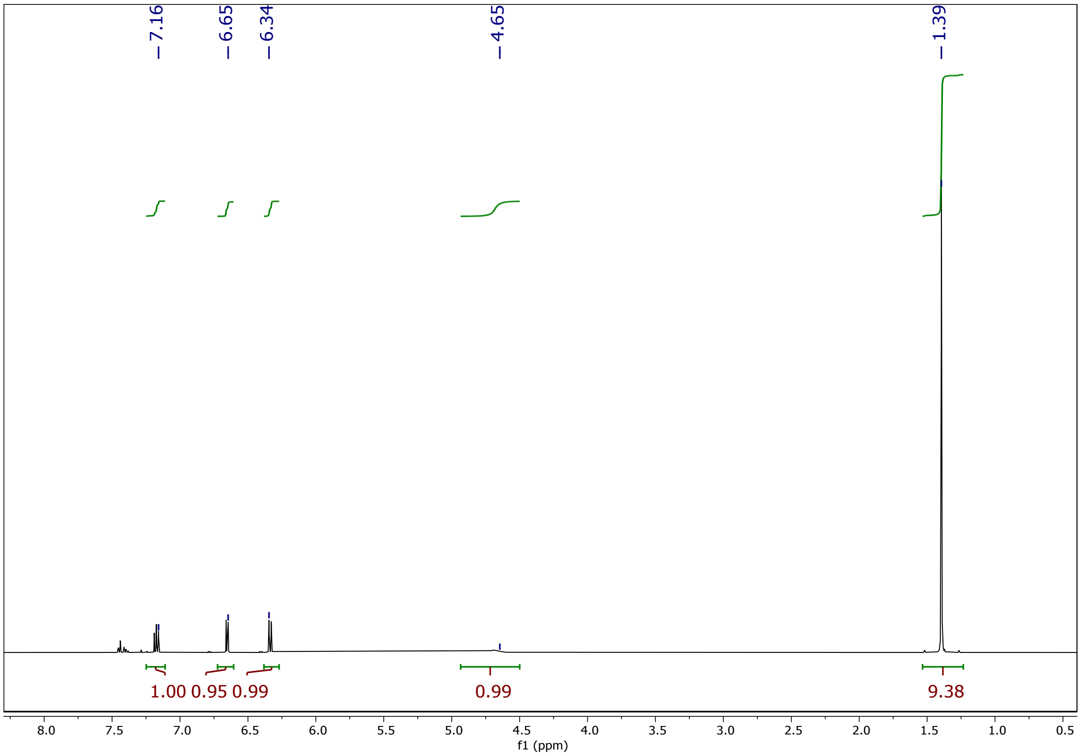

Figure S9. ^{1}H NMR spectrum of **Am5** in $CDCl_3$.

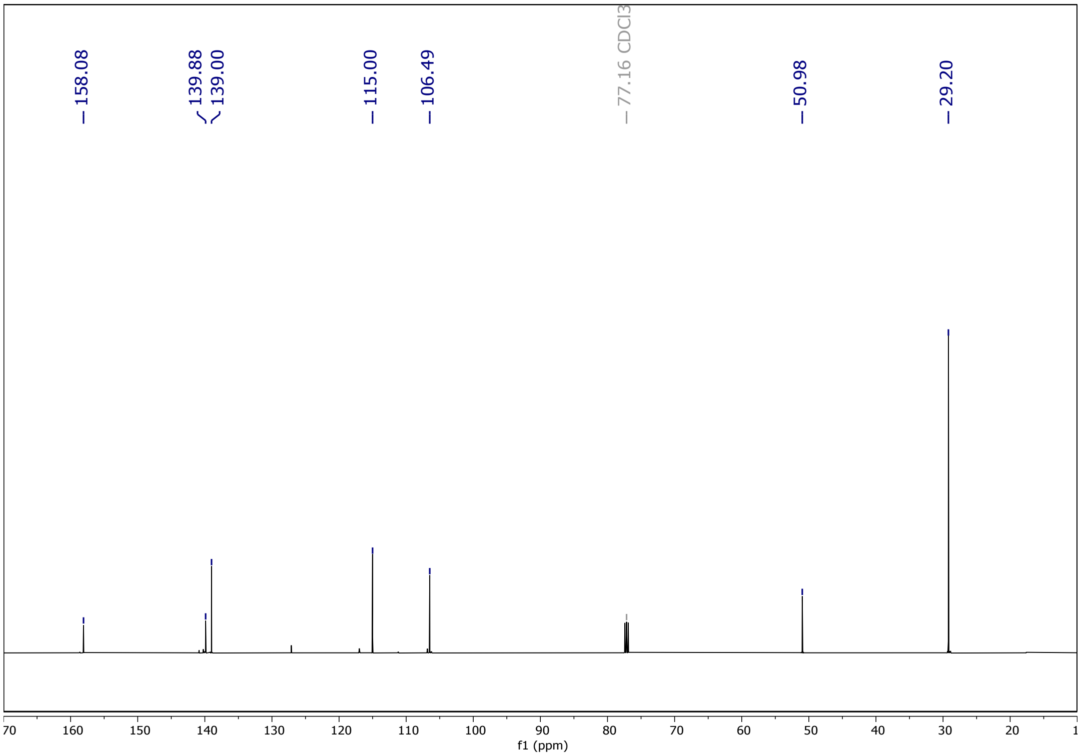

Figure S10. ^{13}C NMR spectrum of **Am5** in $CDCl_3$.

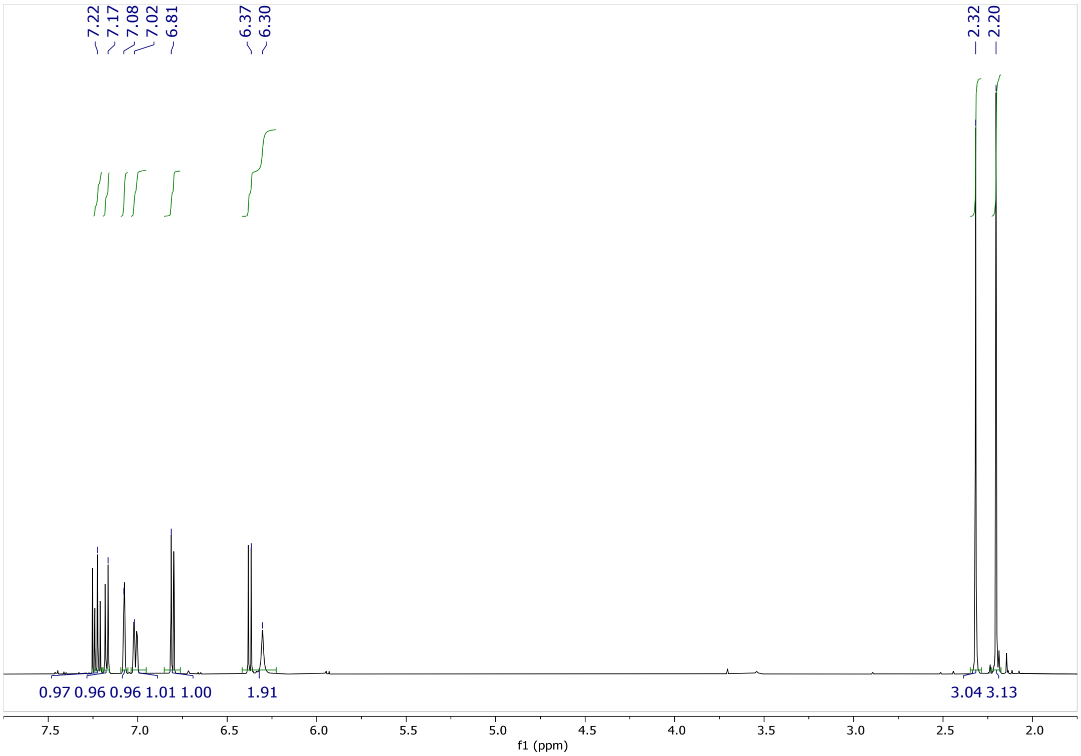

Figure S11. ^{1}H NMR spectrum of **Am7** in $CDCl_3$.

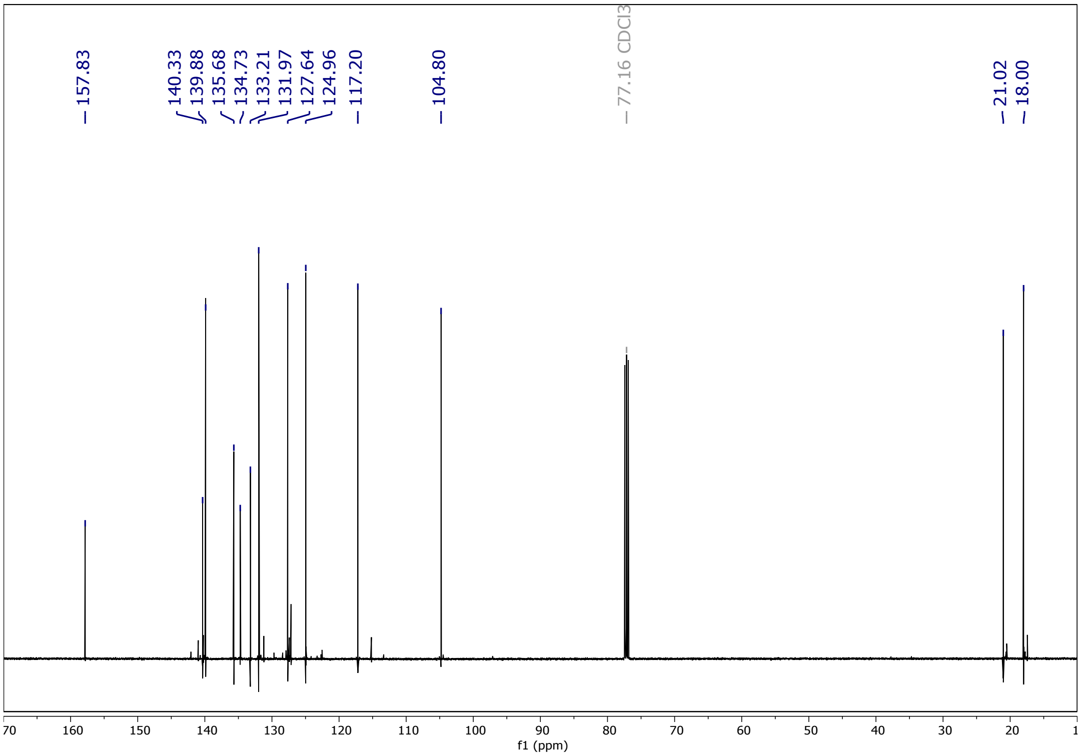

Figure S12. ^{13}C NMR spectrum of **Am7** in $CDCl_3$.

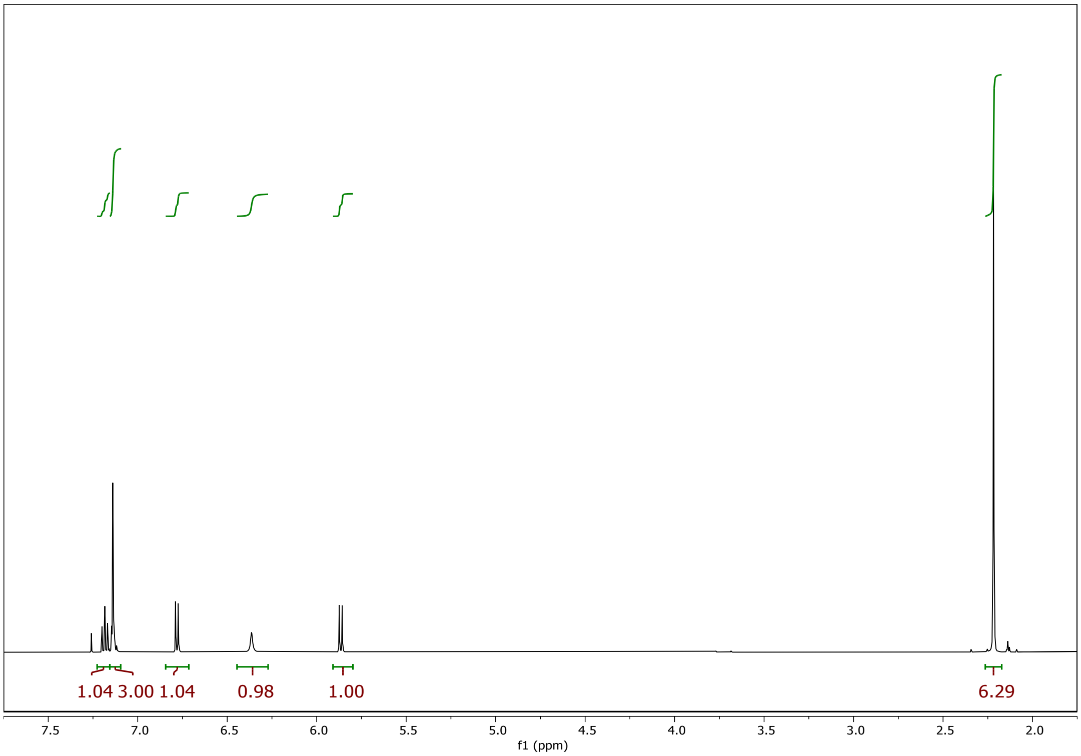

Figure S13. ^{1}H NMR spectrum of **Am8** in $CDCl_3$.

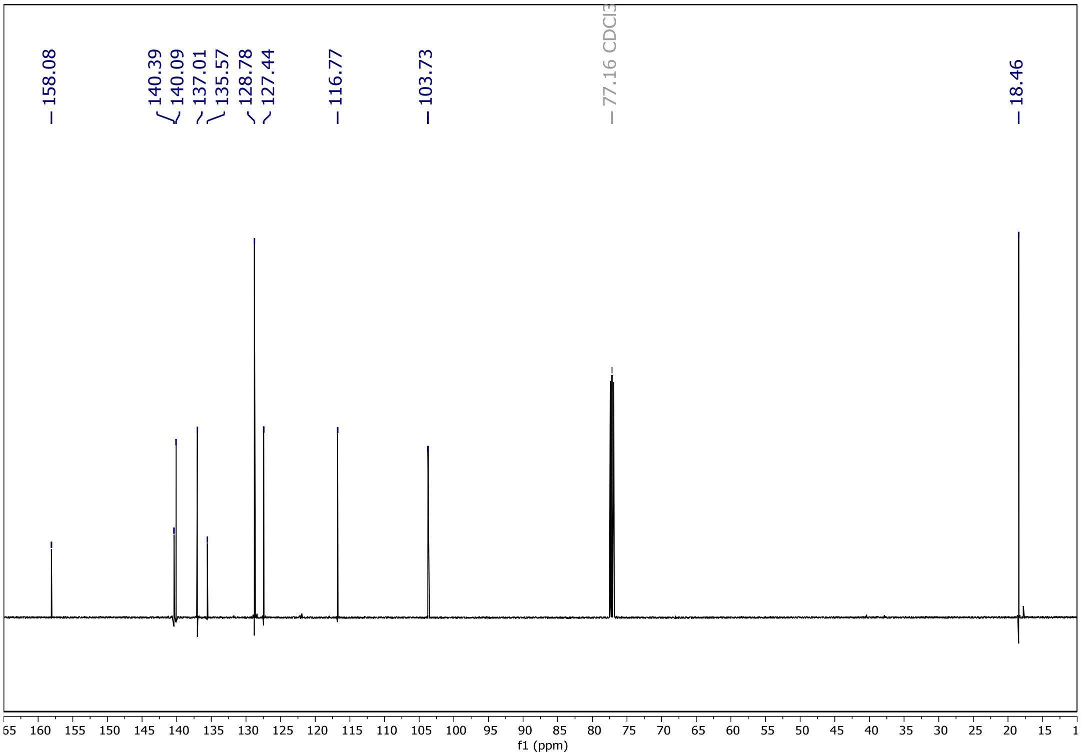

Figure S14. ^{13}C NMR spectrum of **Am8** in $CDCl_3$.

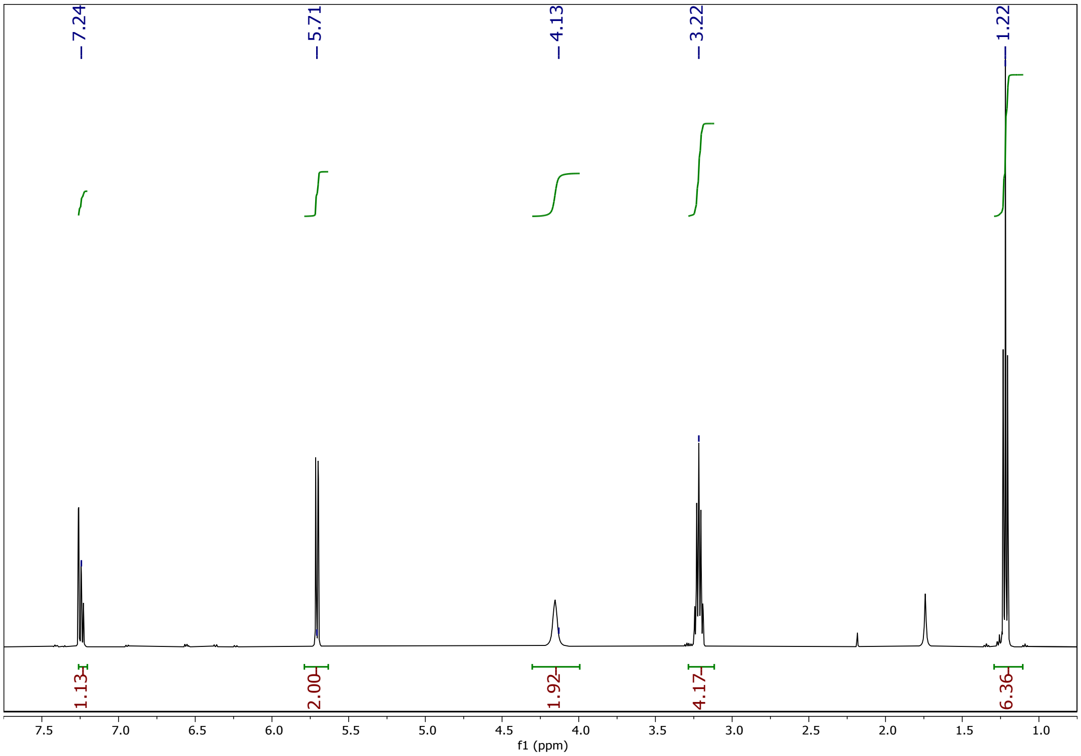

Figure S15. ^{1}H NMR spectrum of **DAm1** in $CDCl_3$.

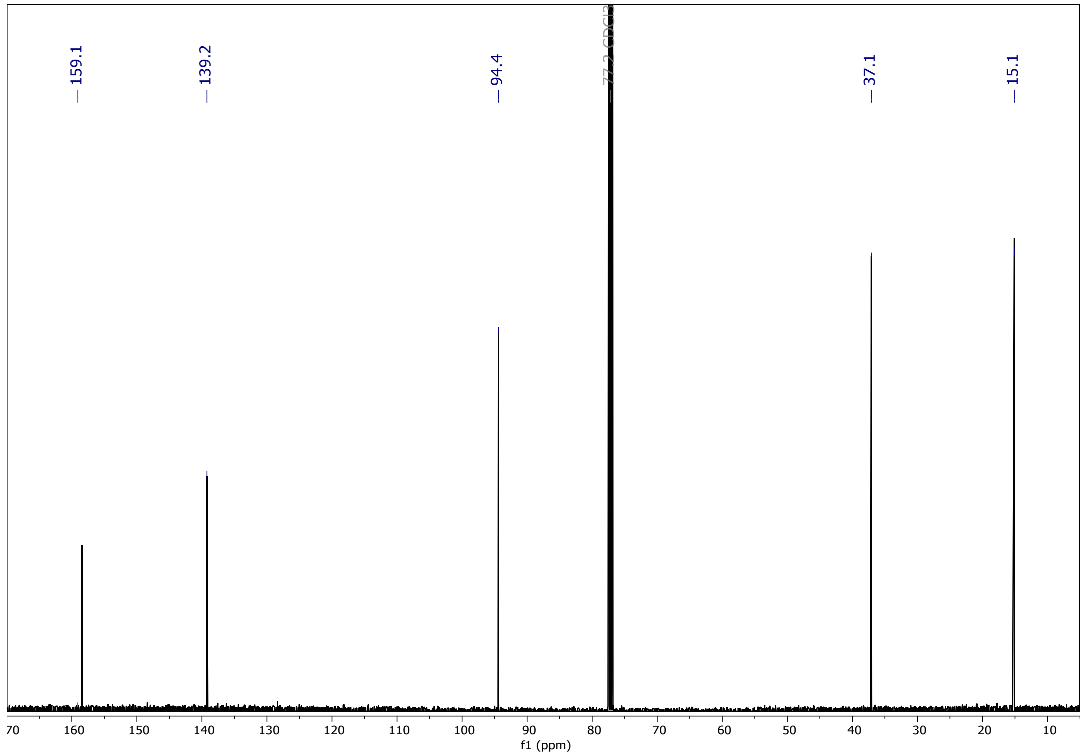

Figure S16. ^{13}C NMR spectrum of **DAm1** in $CDCl_3$.

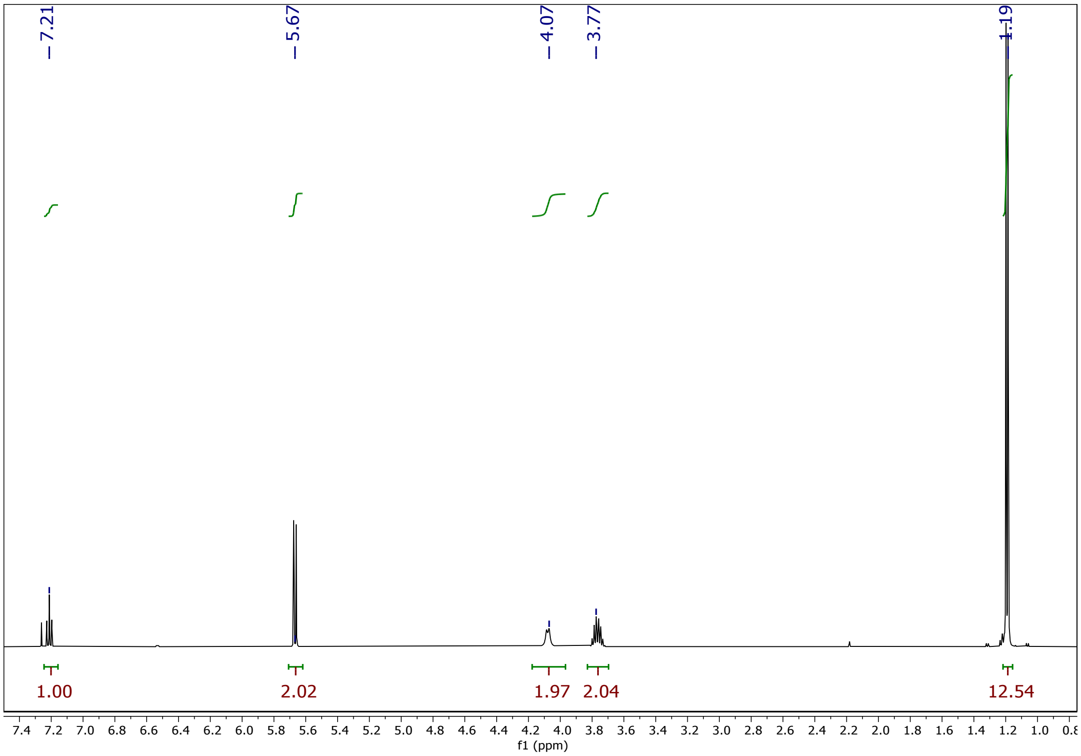

Figure S17. ^{1}H NMR spectrum of **DAm2** in $CDCl_3$.

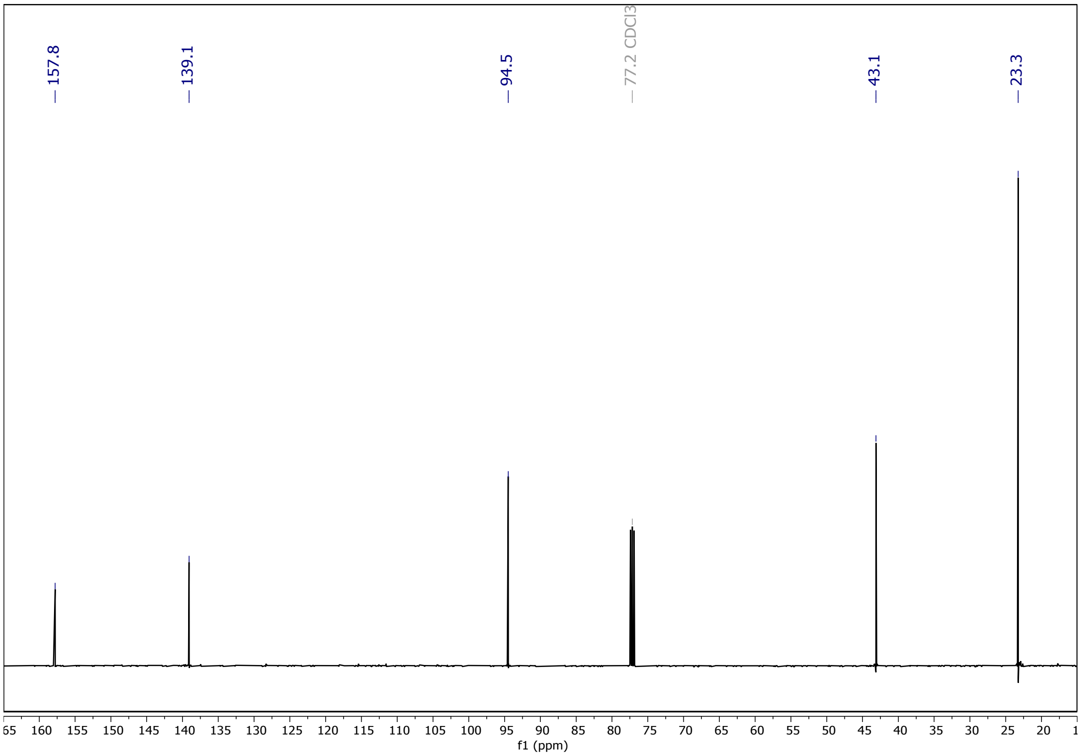

Figure S18. ^{13}C NMR spectrum of **DAm2** in $CDCl_3$.

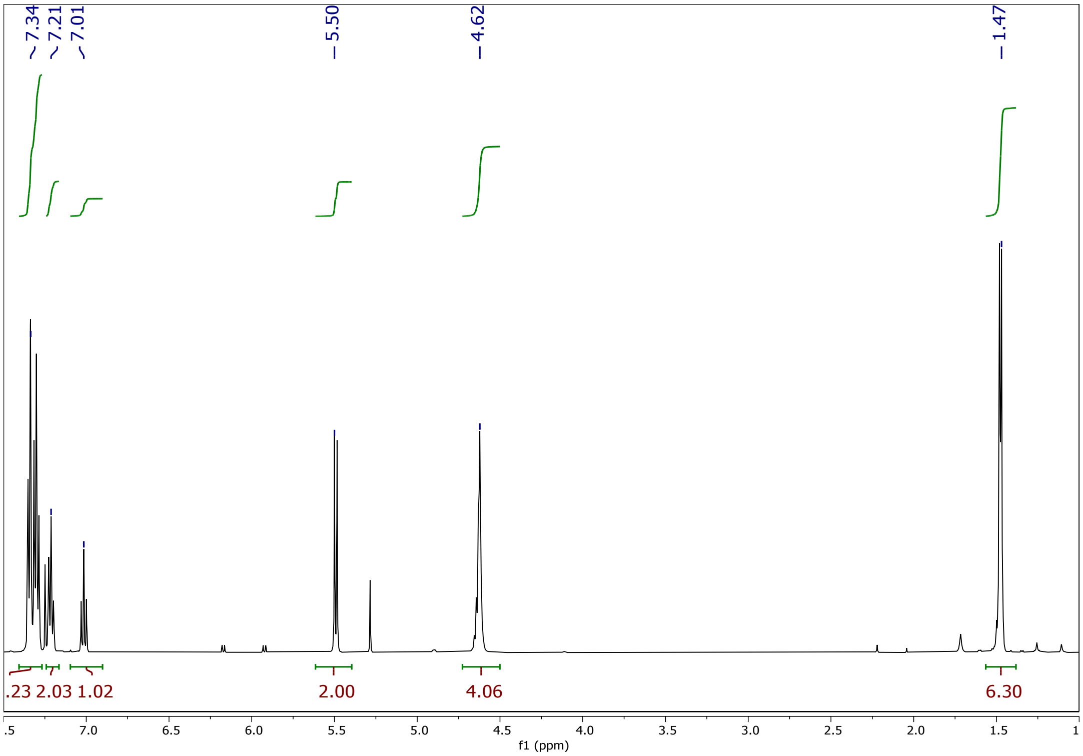

Figure S19. ^{1}H NMR spectrum of **DAm3** in $CDCl_3$. Dichloromethane impurity at 5.30 ppm.

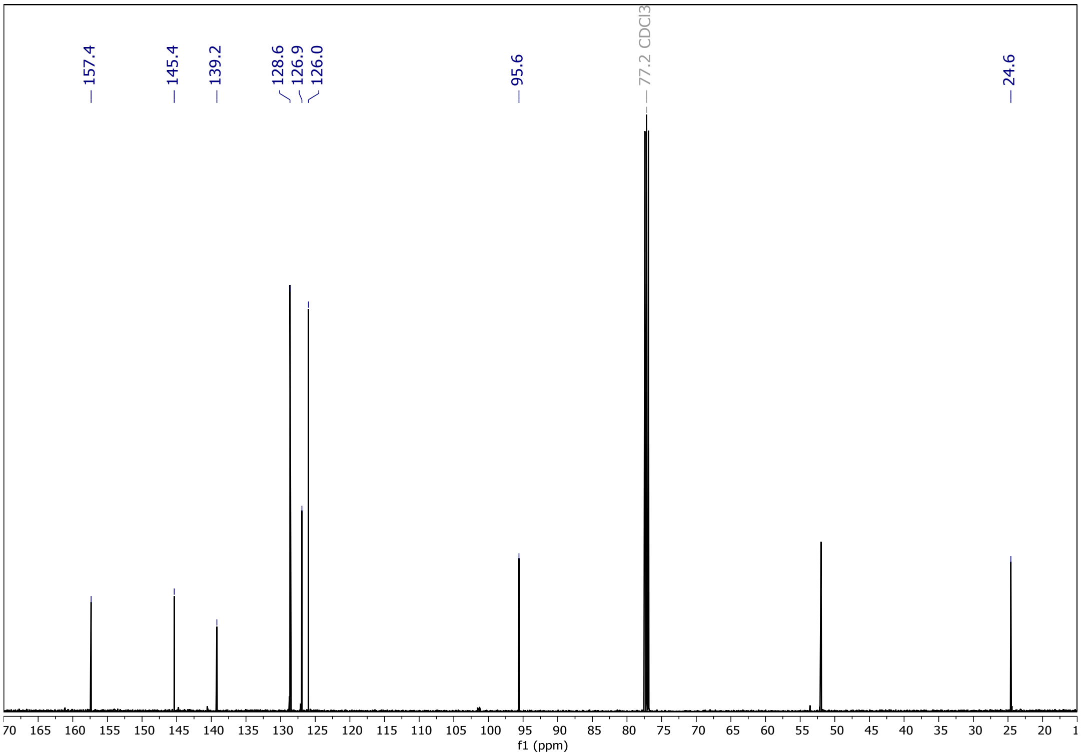

Figure S20. ^{13}C NMR spectrum of **DAm3** in $CDCl_3$. Dichloromethane impurity at 53.52 ppm.

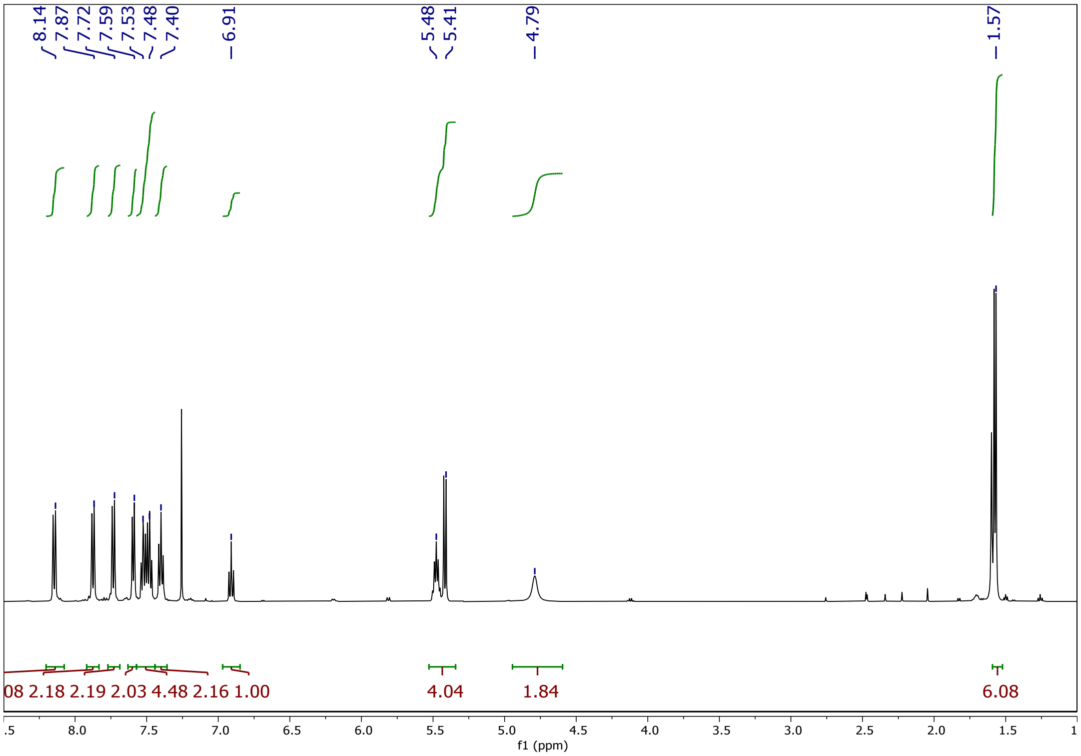

Figure S21. ^{1}H NMR spectrum of **DAm4** in $CDCl_3$.

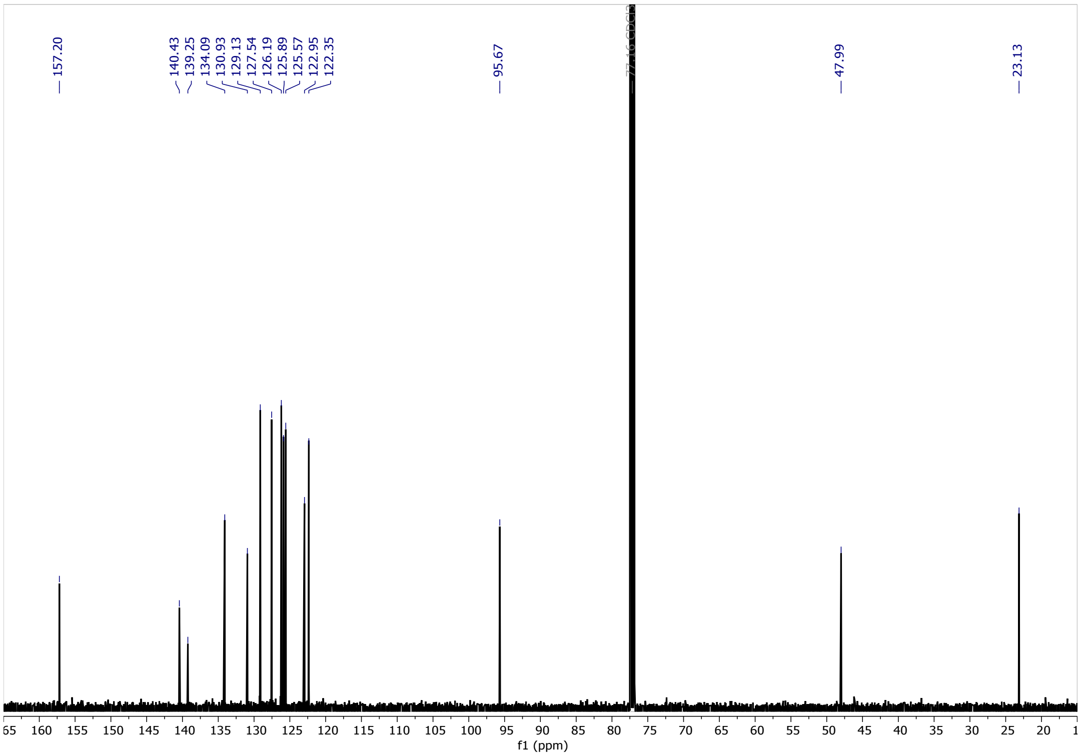

Figure S22. ^{13}C NMR spectrum of **DAm4** in $CDCl_3$.

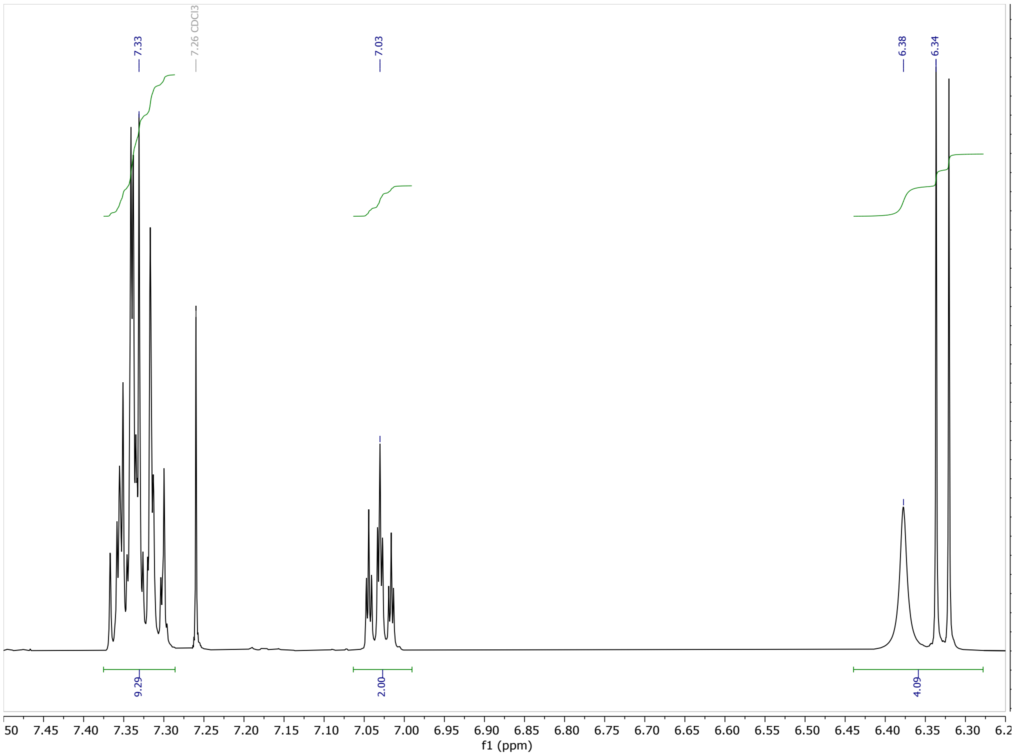

Figure S23. ^{1}H NMR spectrum of **DAm6** in $CDCl_3$.

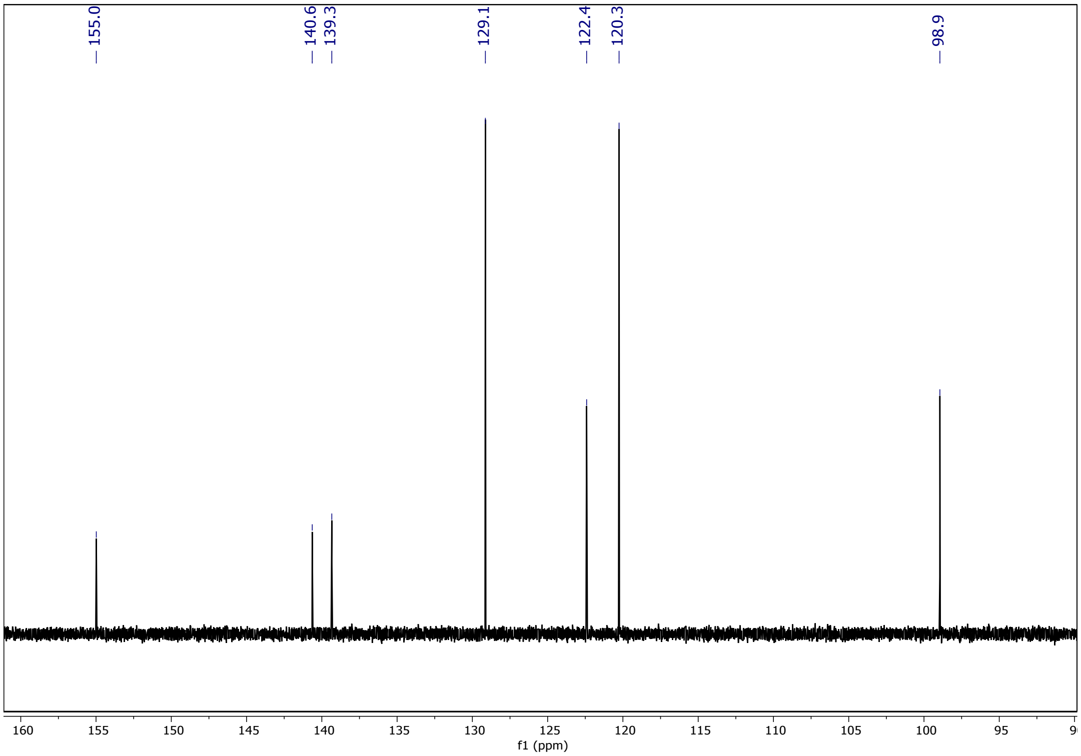

Figure S24. ^{13}C NMR spectrum of **DAm6** in $CDCl_3$.

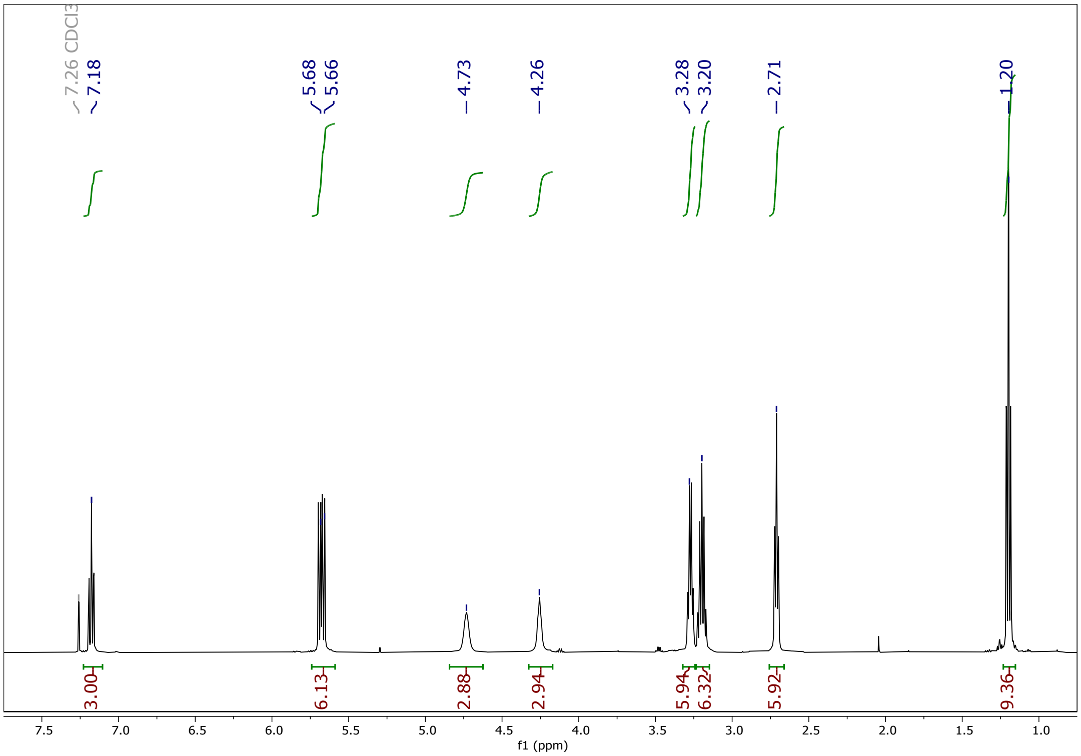

Figure S25. ^{1}H NMR spectrum of **TrAm1** in $CDCl_3$.

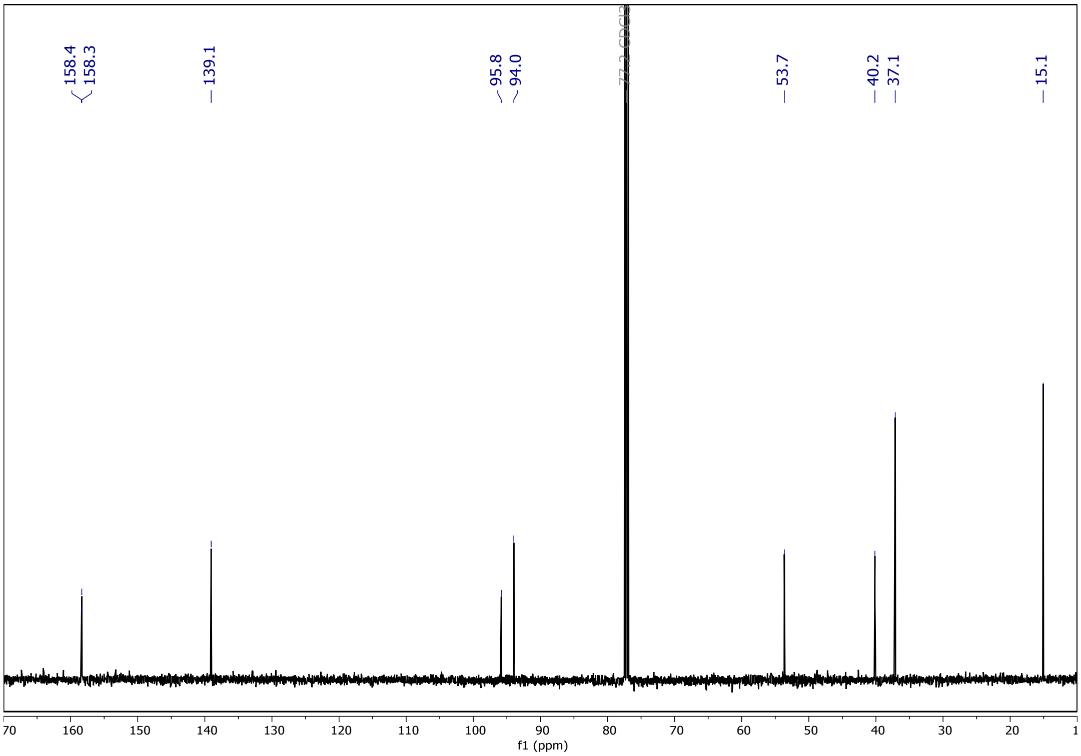

Figure S26. ^{13}C NMR spectrum of **TrAm1** in $CDCl_3$.

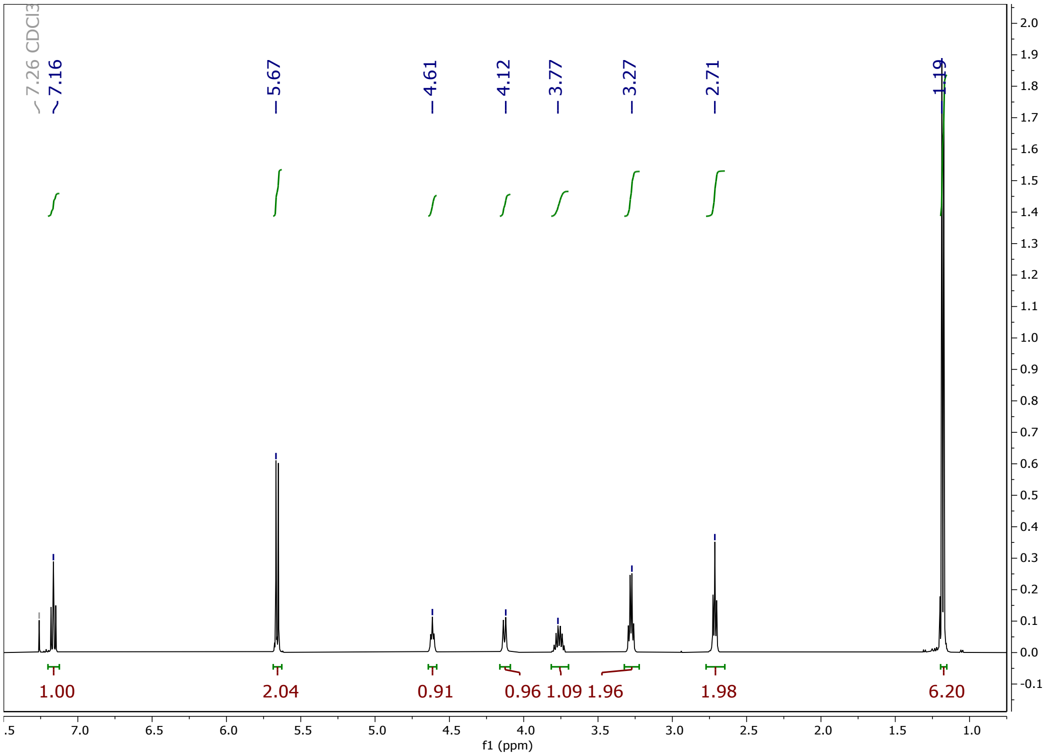

Figure S27. ^{1}H NMR spectrum of **TrAm2** in $CDCl_3$.

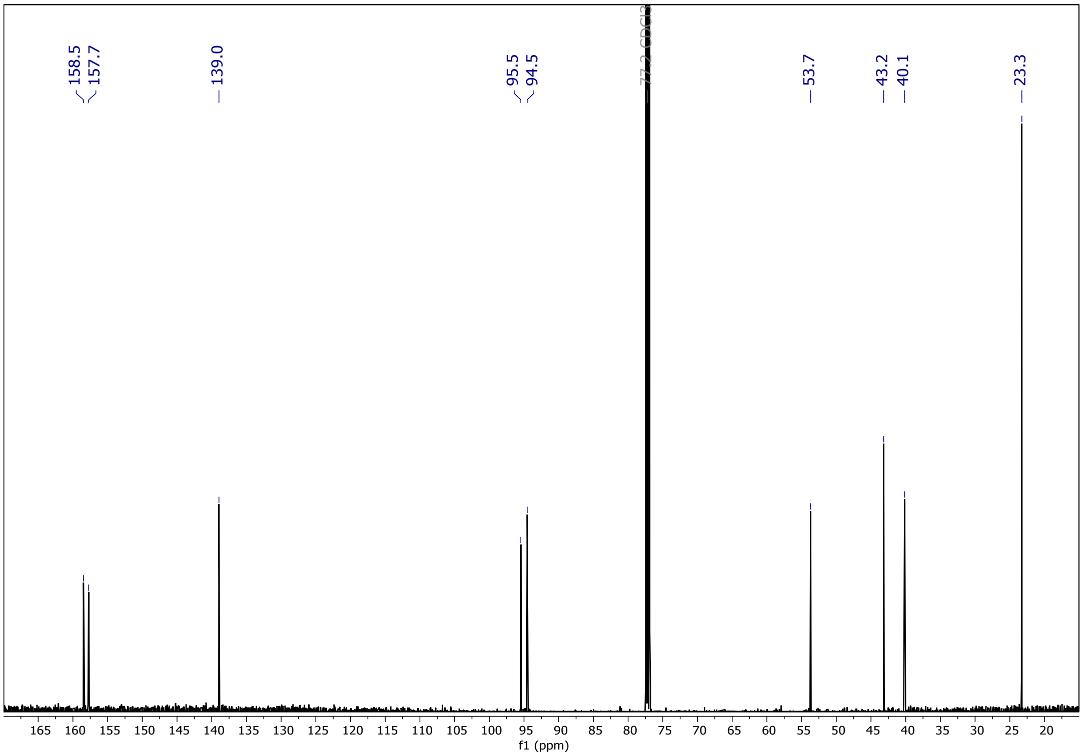

Figure S28. ^{13}C NMR spectrum of **TrAm2** in $CDCl_3$.

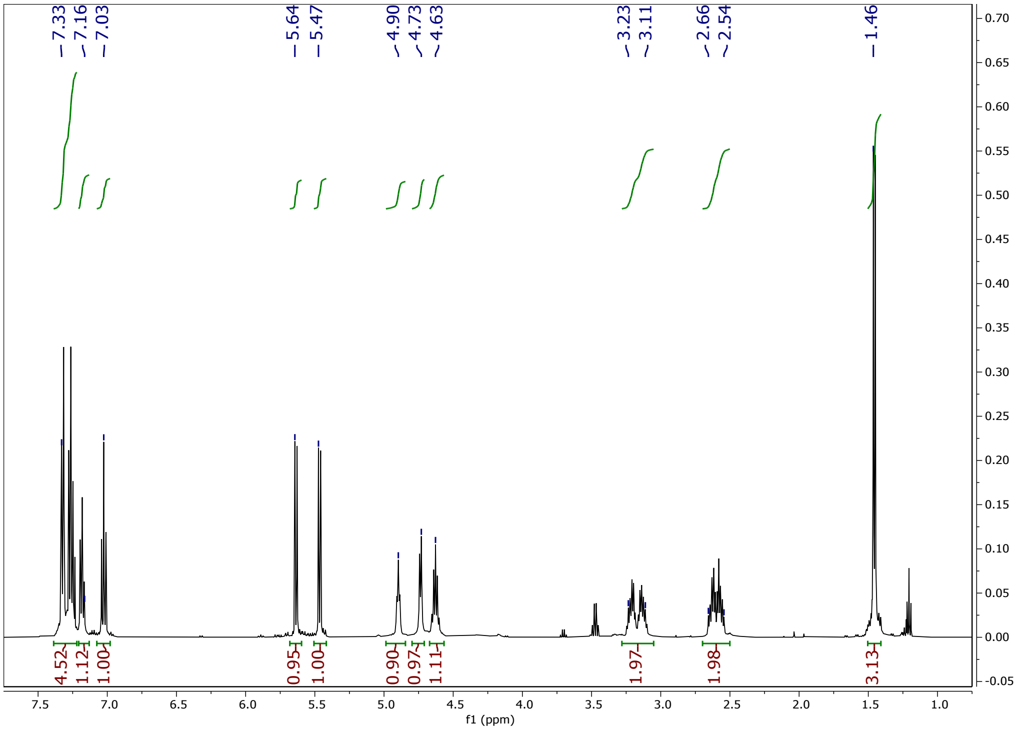

Figure S29. ^{1}H NMR spectrum of **TrAm3** in $CDCl_3$. Peaks at 1.21 and 3.48 ppm are a diethyl ether impurity.

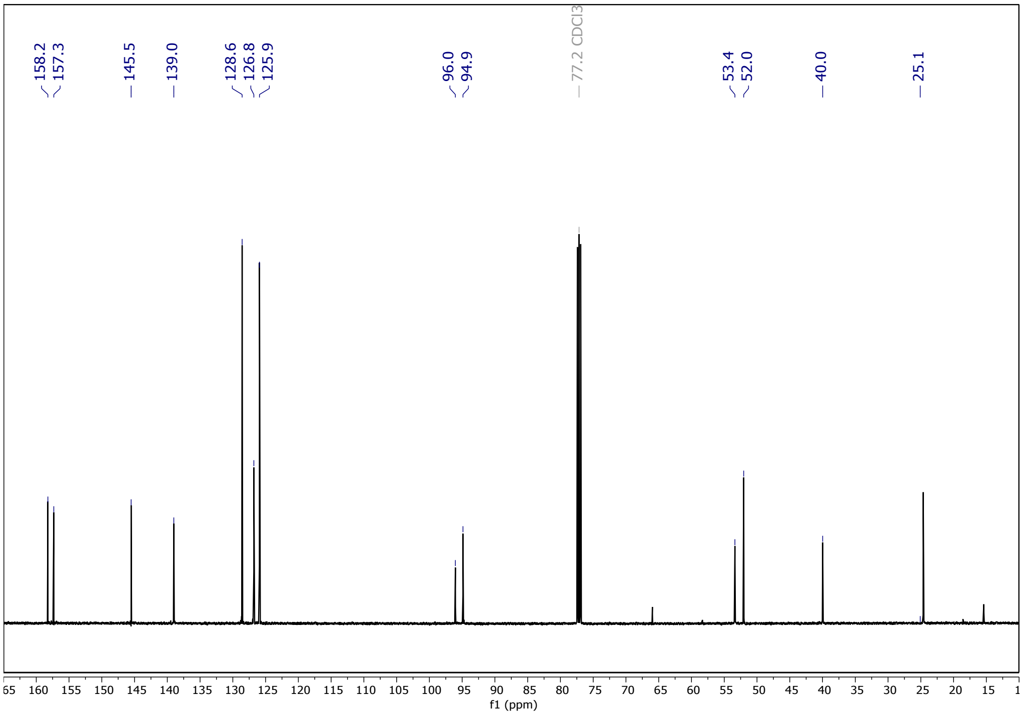

Figure S30. ^{13}C NMR spectrum of **TrAm3** in $CDCl_3$. Peaks at 15.2 and 65.9 ppm are a diethyl ether impurity.

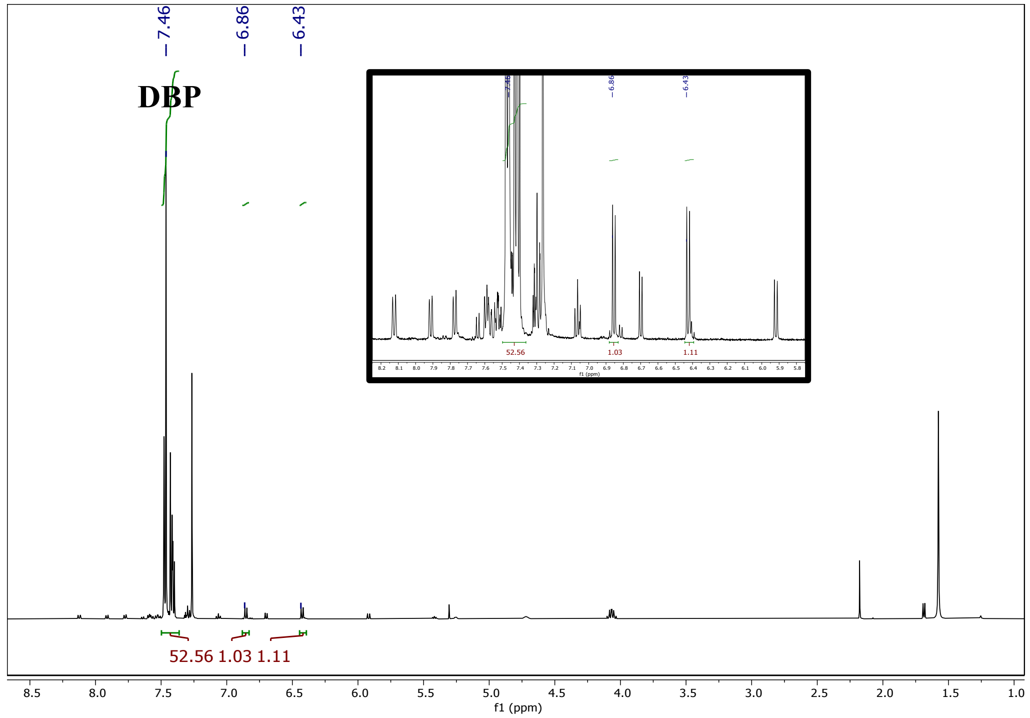

Figure S31. ^{1}H NMR spectra of residue of reaction between 2,6-dibromopyridine (DBP) and 2,2,2-trifluoroethylamine. Inset highlights the region from 5.75 to 8.25 ppm.

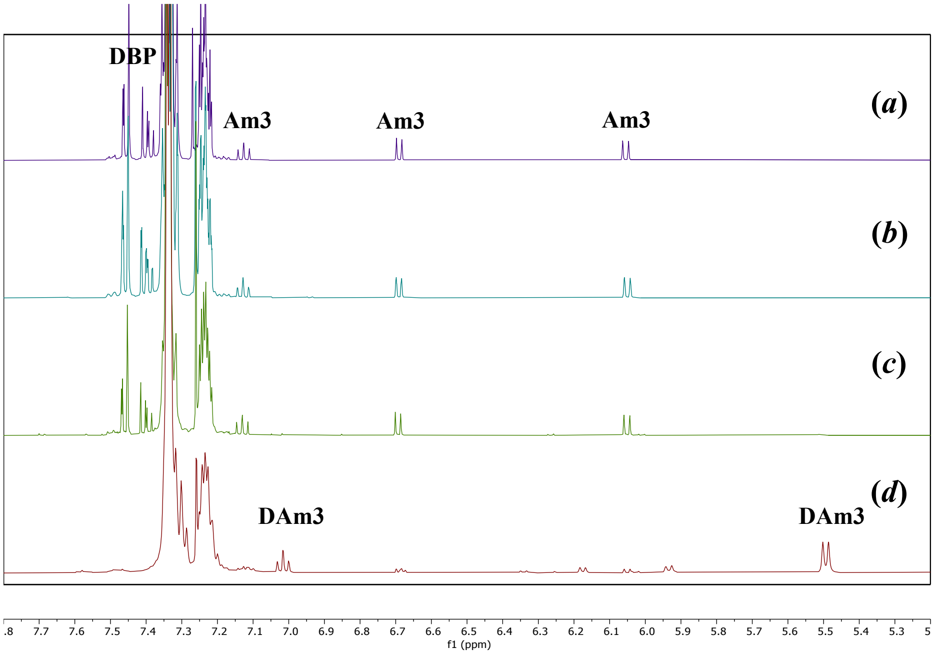

Figure S32. ^{1}H NMR spectra comparing the presence or absence of CuI and DMPAO in the synthesis of **DAm3** at 140 °C for 45 minutes under microwave irradiation, (*a*) No CuI or DMPAO, (*b*) No CuI, (*c*) No DMPAO, and (*d*) no deviation from standard conditions. DBP above refers to signals originating from 2,6-dibromopyridine.

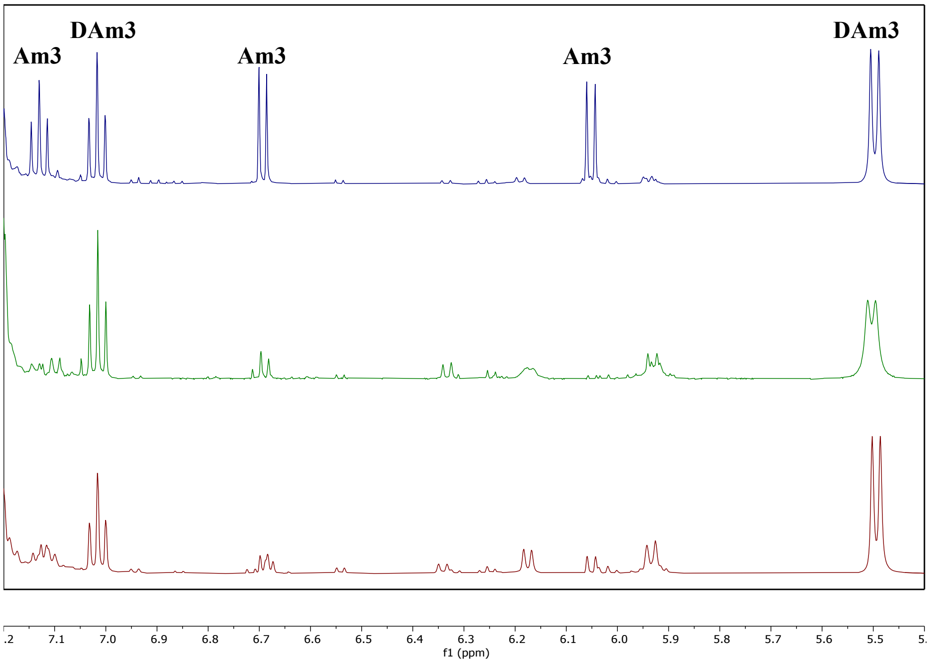

Figure S33. ^{1}H NMR spectra comparing the synthesis of **DAm3** at 140 °C for 45 minutes under microwave irradiation with (*top*) 0 eq, (*middle*) 2 eq, or (*bottom*) 4 eq of K_2CO_3.

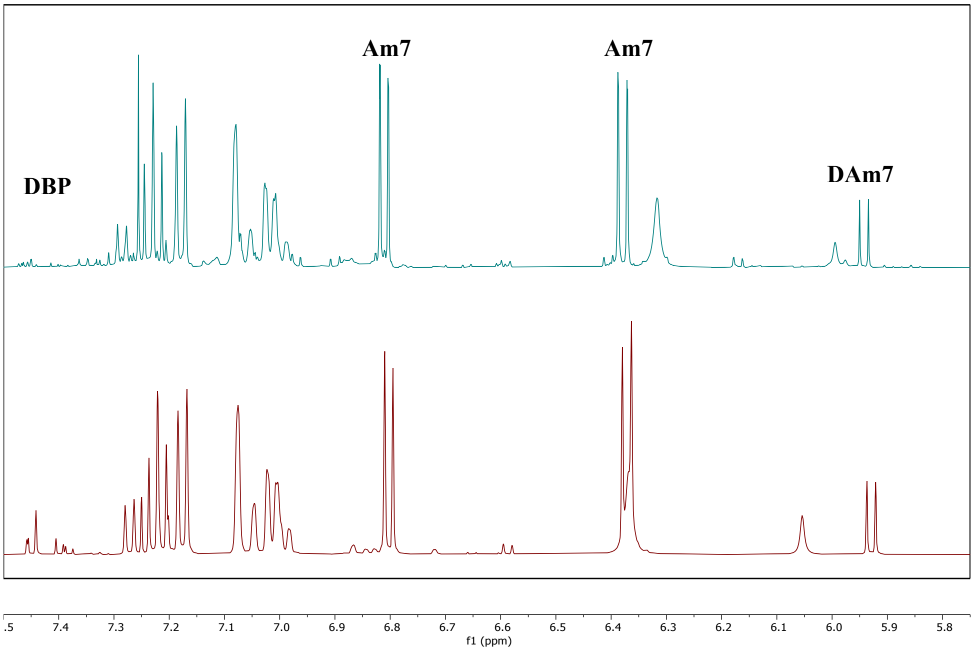

Figure S34. ^{1}H NMR spectra comparing Cu/DMPAO catalyzed (*top*) and uncatalyzed (*bottom*) reactions of 2,4-dimethylaniline and DBP after removal of residual 2,4-dimethylaniline. Both reactions were run at 190 °C for 2.5 hr.

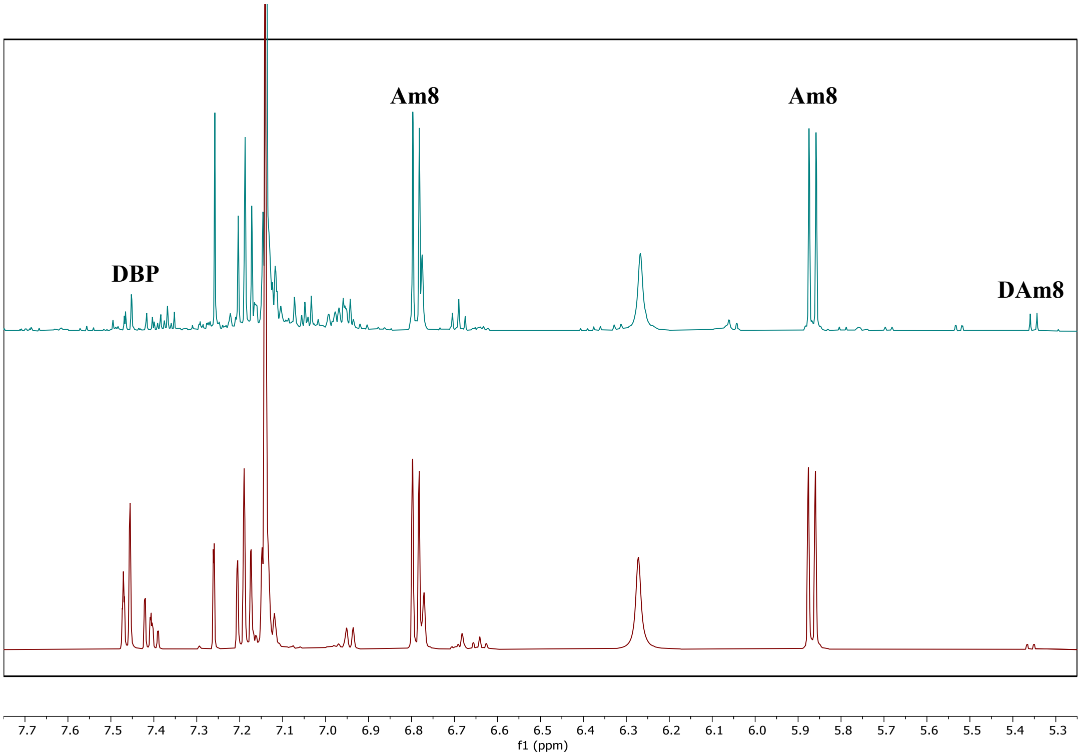

Figure S35. ^{1}H NMR spectra comparing Cu/DMPAO catalyzed (*top*) and uncatalyzed (*bottom*) reactions of 2,6-dimethylaniline and DBP after removal of residual 2,6-dimethylaniline. Both reactions were run at 190 °C for 2.5 hr.

IV. IR Spectra

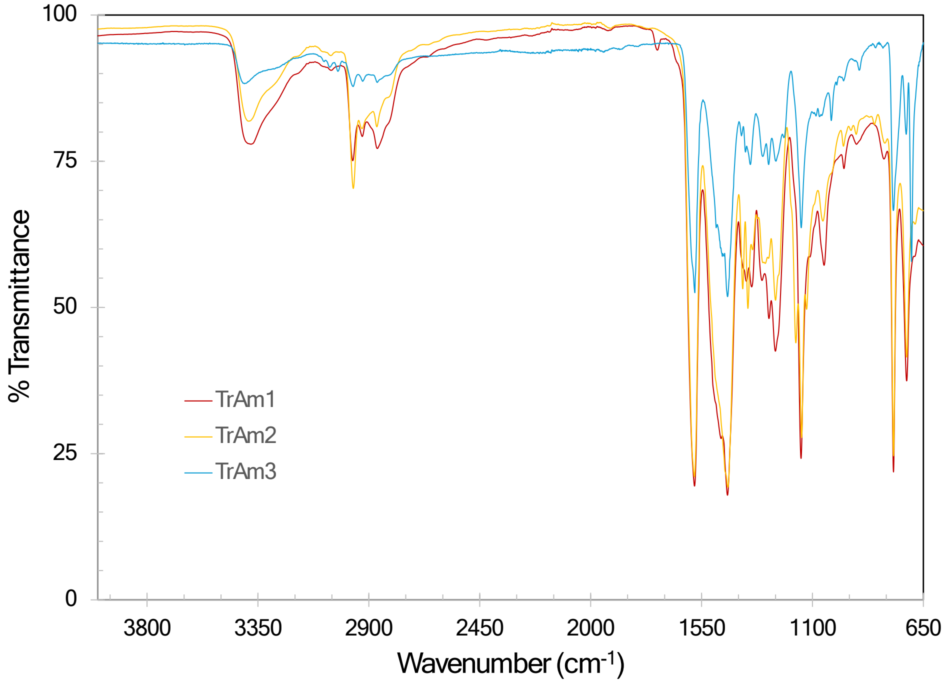

Figure S36. Infrared spectra of **TrAm1-3** collected as solids on an ATR stage.

V. High-Res Mass Spectra

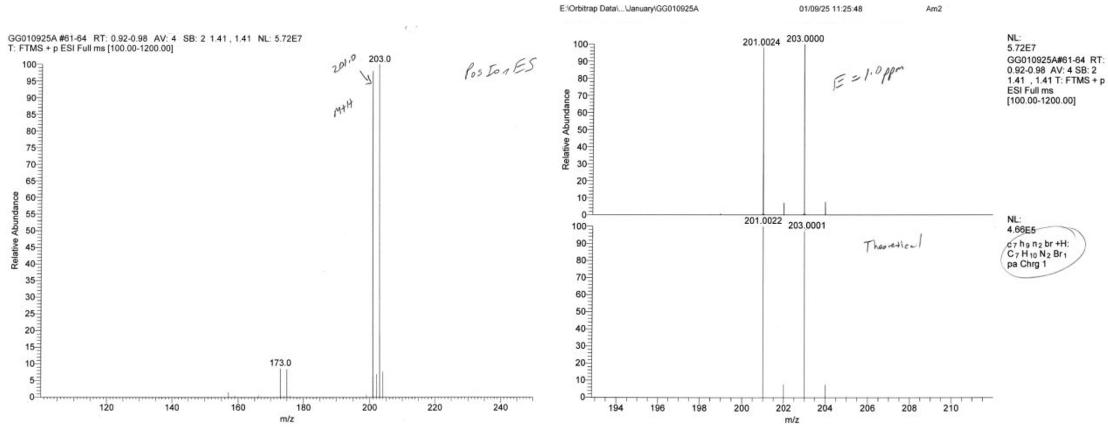

Figure S37 Full mass spectrum for **Am1** (*left*) and high-res peak measurement of $[M+H]^+$ peak with comparison to theoretical. (*right*)

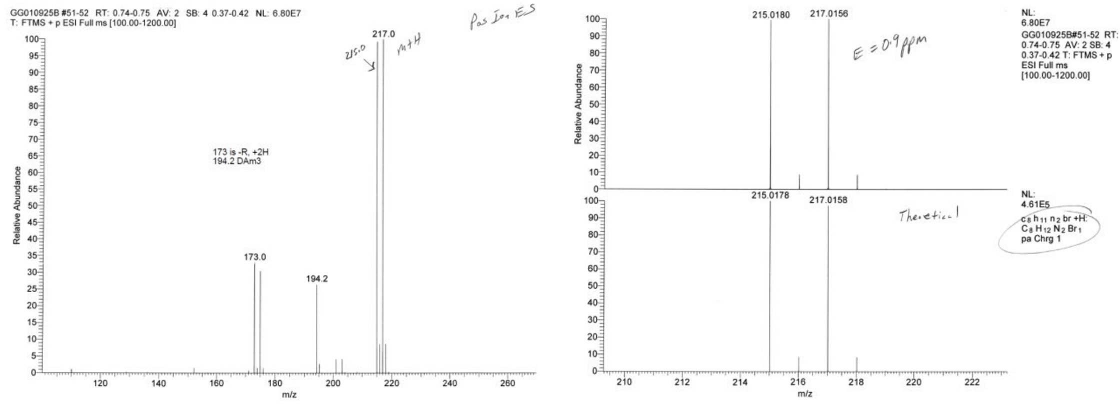

Figure S38 Full mass spectrum for **Am2** (*left*) and high-res peak measurement of $[M+H]^+$ peak with comparison to theoretical. (*right*)

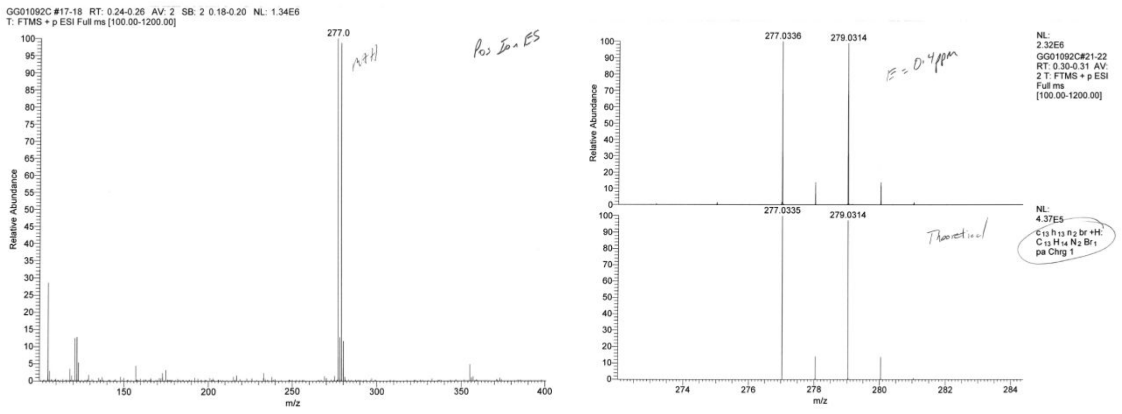

Figure S39 Full mass spectrum for **Am3** (*left*) and high-res peak measurement of $[M+H]^+$ peak with comparison to theoretical. (*right*)

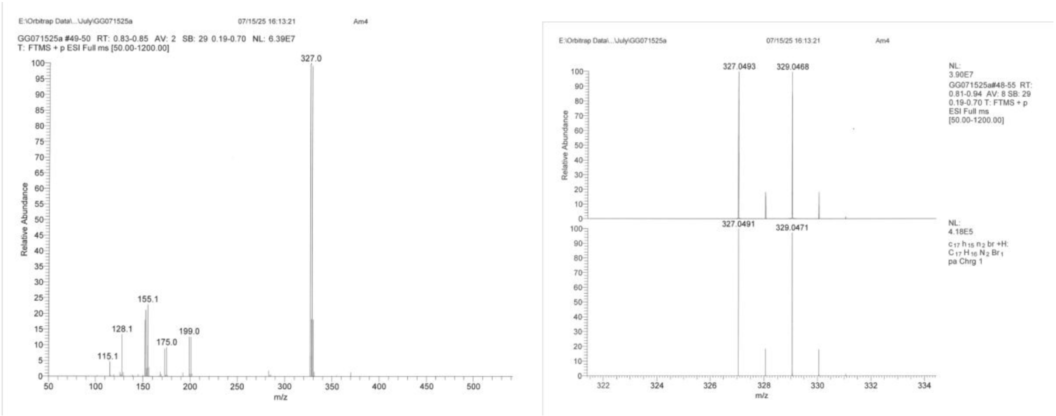

Figure S40 Full mass spectrum for **Am4** (*left*) and high-res peak measurement of $[M+H]^+$ peak with comparison to theoretical. (*right*)

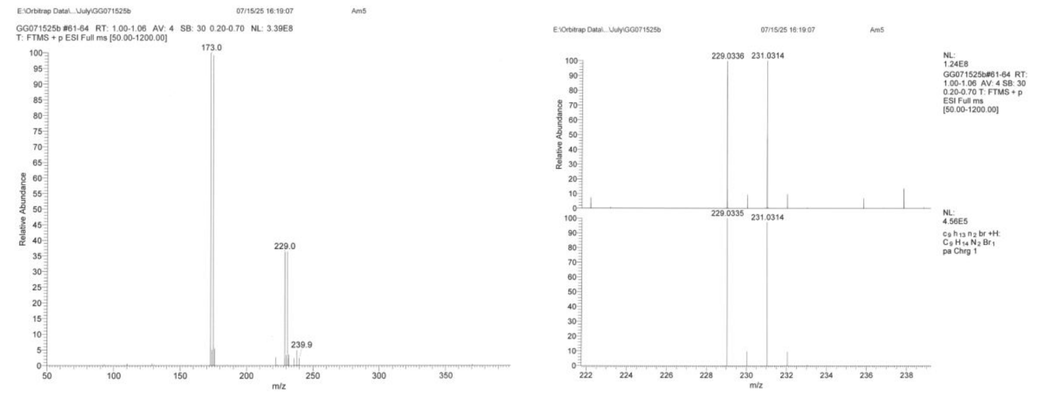

Figure S41 Full mass spectrum for **Am5** (*left*) and high-res peak measurement of $[M+H]^+$ peak with comparison to theoretical. (*right*)

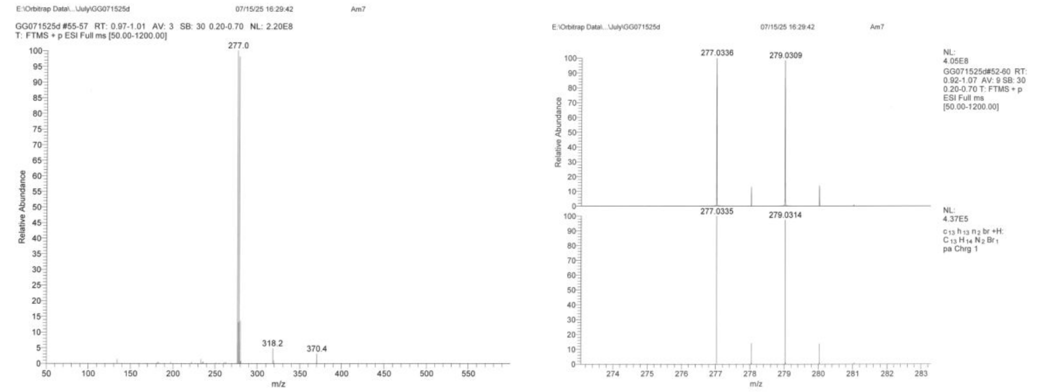

Figure S42 Full mass spectrum for **Am7** (*left*) and high-res peak measurement of $[M+H]^+$ peak with comparison to theoretical. (*right*)

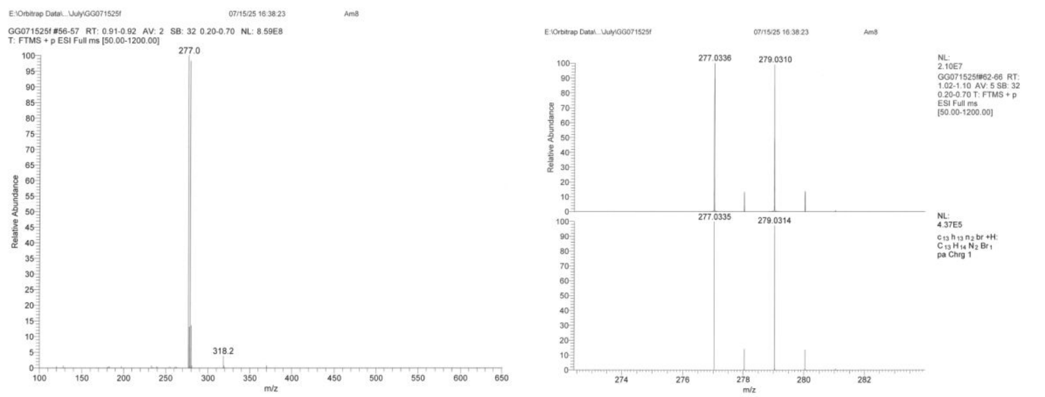

Figure S43 Full mass spectrum for **Am8** (*left*) and high-res peak measurement of $[M+H]^+$ peak with comparison to theoretical. (*right*)

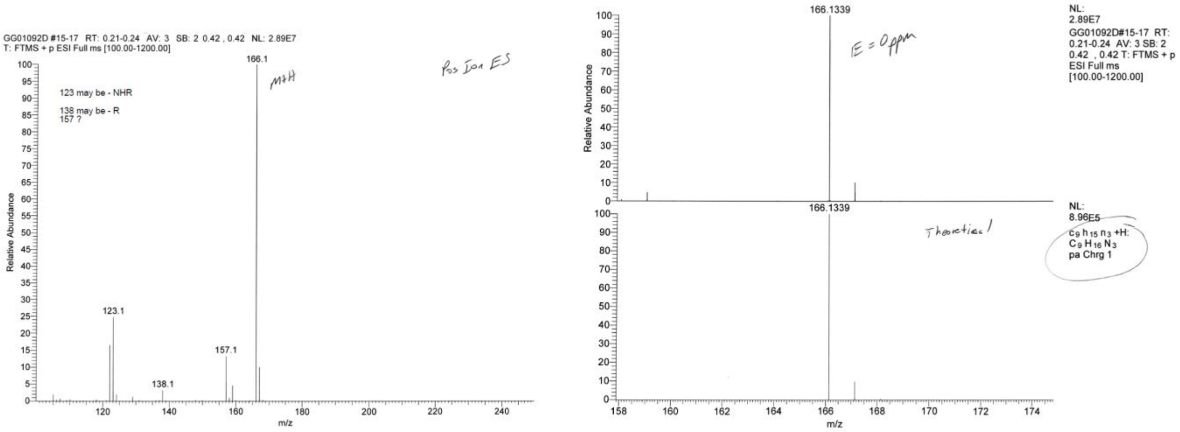

Figure S44 Full mass spectrum for **DAm1** (*left*) and high-res peak measurement of $[M+H]^+$ peak with comparison to theoretical. (*right*)

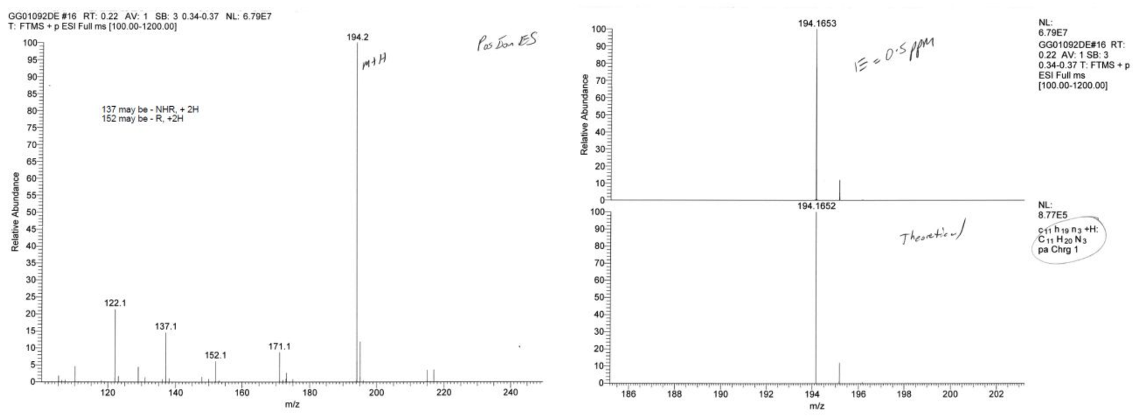

Figure S45 Full mass spectrum for **DAm2** (*left*) and high-res peak measurement of $[M+H]^+$ peak with comparison to theoretical. (*right*)

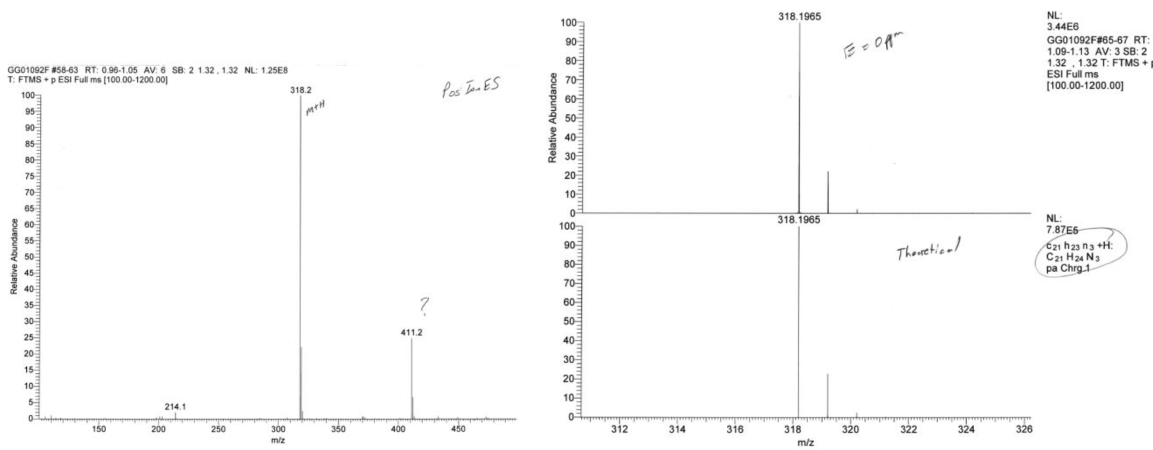

Figure S46 Full mass spectrum for **DAm3** (*left*) and high-res peak measurement of $[M+H]^+$ peak with comparison to theoretical. (*right*)

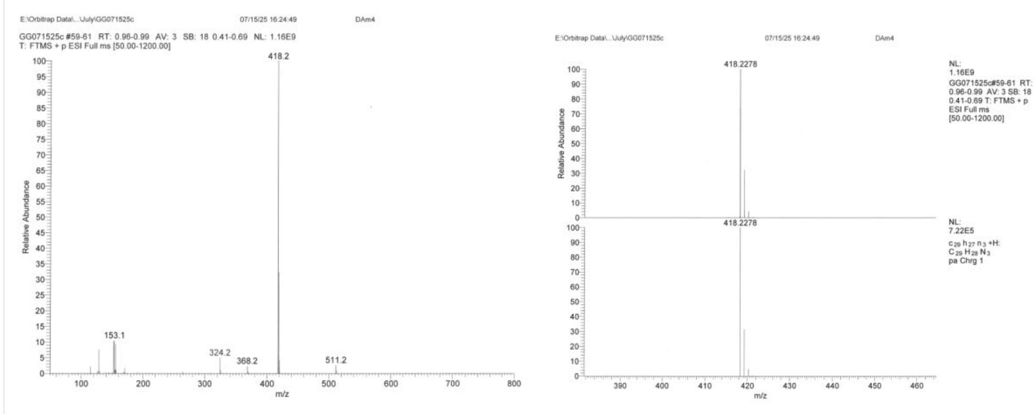

Figure S47 Full mass spectrum for **DAm4** (*left*) and high-res peak measurement of $[M+H]^+$ peak with comparison to theoretical. (*right*)

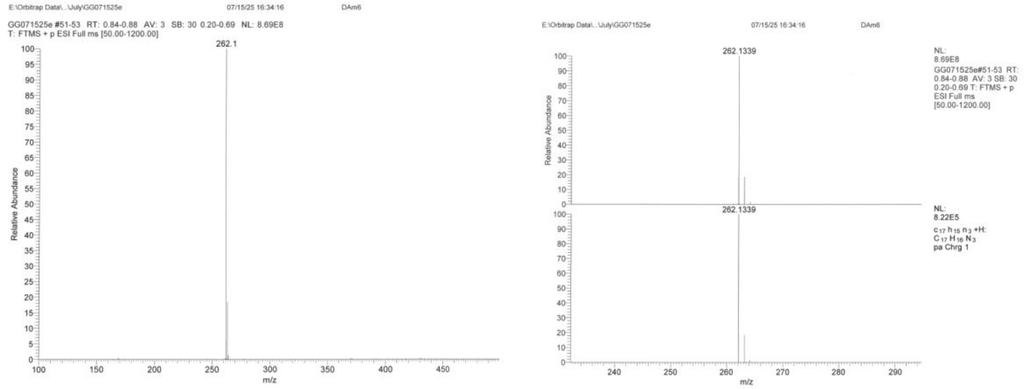

Figure S48 Full mass spectrum for **DAm6** (*left*) and high-res peak measurement of $[M+H]^+$ peak with comparison to theoretical. (*right*)

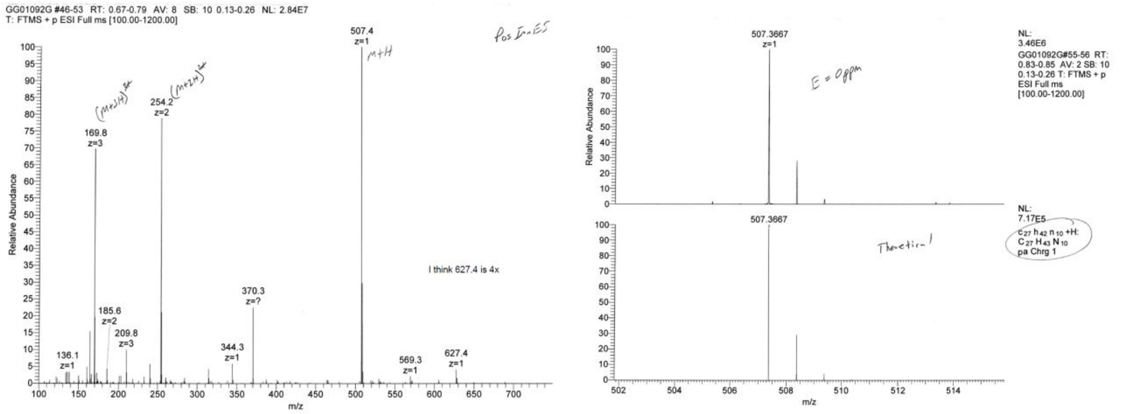

Figure S49 Full mass spectrum for **TrAm1** (*left*) and high-res peak measurement of $[M+H]^+$ peak with comparison to theoretical. (*right*)

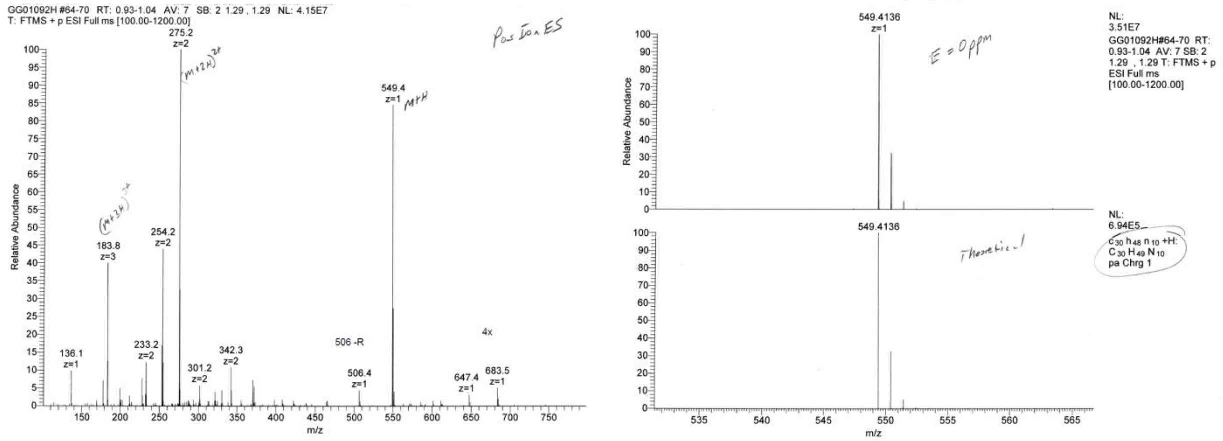

Figure S50 Full mass spectrum for **TrAm2** (*left*) and high-res peak measurement of $[M+H]^+$ peak with comparison to theoretical. (*right*)

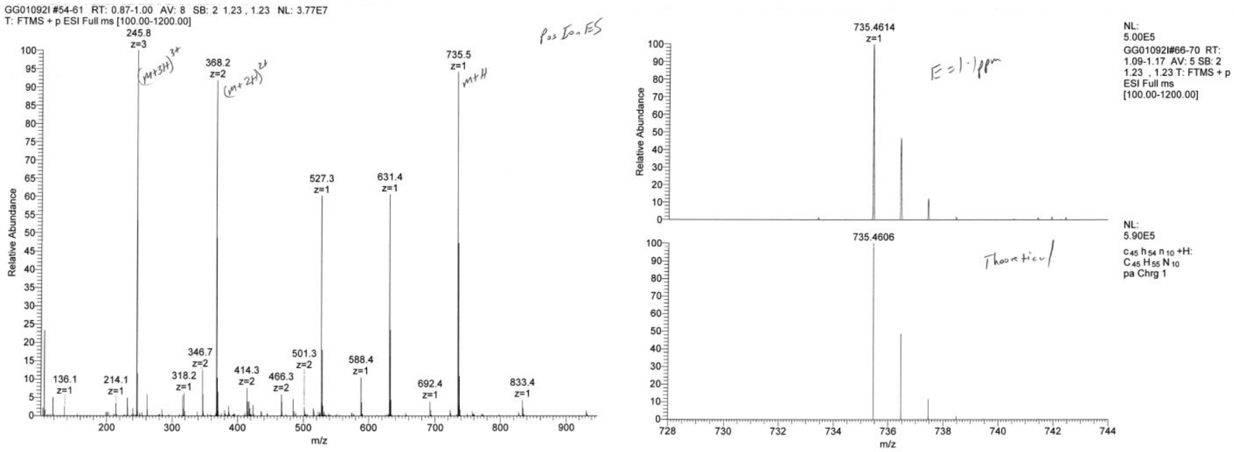

Figure S51 Full mass spectrum for **TrAm3** (*left*) and high-res peak measurement of $[M+H]^+$ peak with comparison to theoretical. (*right*)

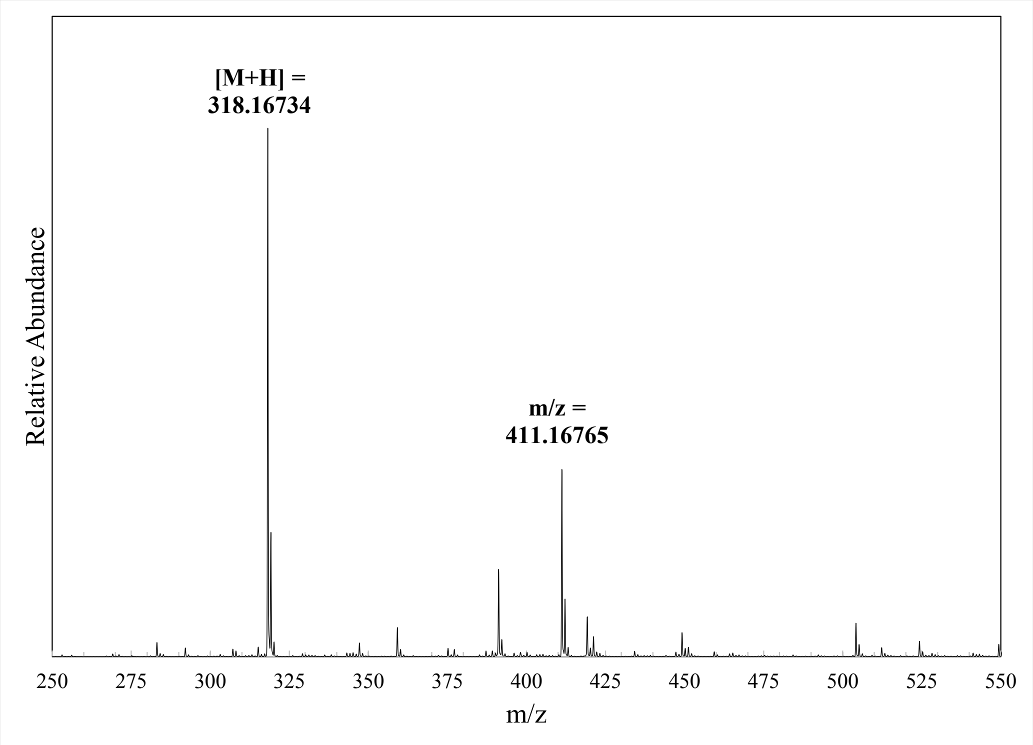

Figure S52 Mass spectrum for a residue from the synthesis of **DAm3**.

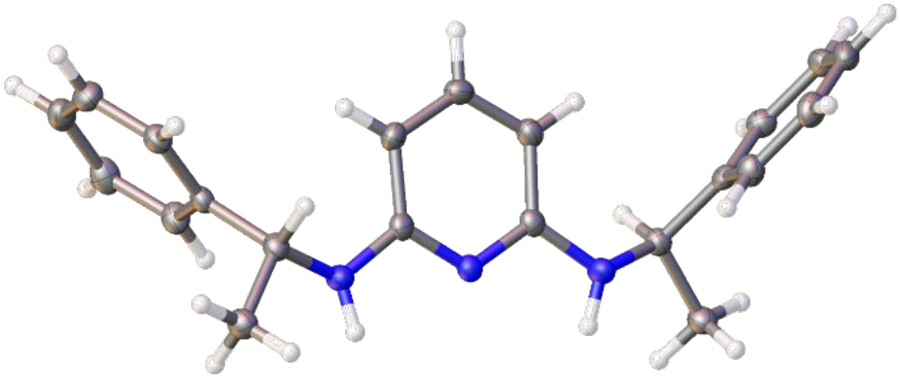

Figure S53 Solid state structure of **DAm3**. Atoms represented as 50% probability ellipsoids. C, N, and H are represented by blue, grey, and white ellipsoids, respectively. All H in calculated positions except for N-H positions which were freely refined.

Table S1 Crystallographic parameters.

	DAm3
Empirical formula	$C_{21}H_{23}N_3$
Fomula Weight	317.42
Temperature (K)	105(7)
Wavelength (Å)	1.54184
Crystal System	Orthorhombic
Space Group	$P2_12_12_1$
a (Å)	5.73911(9)
b (Å)	16.1525(3)
c (Å)	19.0100(3)
α (°)	90
β (°)	90
γ (°)	90
Volume(Å^3)	1762.25(5)
Z	4
ρ_{calc}g/cm^3	1.196
μ/mm^{-1}	0.55
F(000)	680
Crystal size/mm^3	0.272 × 0.159 × 0.105
Radiation	Cu Kα (λ = 1.54184)
2Θ range for data collection/°	7.182 to 139.11
Index ranges	$-6 \leq h \leq 6$, $-19 \leq k \leq 18$, $-18 \leq l \leq 23$
Reflections collected	8186
Independent reflections	2959 [R_{int} = 0.0226, R_{sigma} = 0.0253]
Data/restraints/parameters	2959/0/227
Goodness-of-fit on F^2	1.066
Final R indexes [I>=2σ (I)]	R_1 = 0.0299, wR_2 = 0.0744
Final R indexes [all data]	R_1 = 0.0317, wR_2 = 0.0756
Largest diff. peak/hole / e Å^{-3}	0.11/-0.16
Flack parameter	-0.1(2)

References

1. Rigaku OD (2023). *CrysAlis PRO*. Rigaku Oxford Diffraction,

2. Yarnton, England; Sheldrick, G. M. (2015). *Acta Cryst.* A**71**, 3–8.

3. Sheldrick, G. M. (2015). *Acta Cryst.* C**71**, 3–8.

4. Dolomanov, O. V., Bourhis, L. J., Gildea, R. J., Howard, J. A. K. & Puschmann, H. (2009). *J. Appl. Cryst.* **42**, 339–341.
